# Biomarker-based risk prediction for the onset of neuroinflammation in X-linked adrenoleukodystrophy

**DOI:** 10.1016/j.ebiom.2023.104781

**Published:** 2023-09-07

**Authors:** Isabelle Weinhofer, Paulus Rommer, Andreas Gleiss, Markus Ponleitner, Bettina Zierfuss, Petra Waidhofer-Söllner, Stéphane Fourcade, Katharina Grabmeier-Pfistershammer, Marie-Christine Reinert, Jens Göpfert, Anne Heine, Hemmo A.F. Yska, Carlos Casasnovas, Verónica Cantarín, Caroline G. Bergner, Eric Mallack, Sonja Forss-Petter, Patrick Aubourg, Annette Bley, Marc Engelen, Florian Eichler, Troy C. Lund, Aurora Pujol, Wolfgang Köhler, Jörn-Sven Kühl, Johannes Berger

**Affiliations:** a Department of Pathobiology of the Nervous System, Center for Brain Research, Medical University of Vienna, Vienna, Austria; b Department of Neurology, Comprehensive Center for Clinical Neurosciences and Mental Health, Medical University of Vienna, Vienna, Austria; c Institute of Clinical Biometrics, Center for Medical Data Science, Medical University of Vienna, Vienna, Austria; d Department of Neuroscience, Centre de Recherche du CHUM, Université de Montréal, Montréal, Canada; e Division of Immune Receptors and T Cell Activation, Institute of Immunology, Center for Pathophysiology, Infectiology and Immunology, Medical University of Vienna, Austria; f Neurometabolic Diseases Laboratory, Bellvitge Biomedical Research Institute (IDIBELL), Barcelona, Catalonia, Spain; g Division of Pediatric Neurology, Department of Pediatrics and Adolescent Medicine, University Medical Center Göttingen, Göttingen, Germany; h Applied Biomarkers and Immunoassays Working Group, NMI Natural and Medical Sciences Institute at the University of Tübingen, Reutlingen, Germany; i Department of Pediatric Neurology, Amsterdam Public Health, Amsterdam University Medical Center, Amsterdam, the Netherlands; j Neuromuscular Unit, Neurology Department, Hospital Universitario Bellvitge, Bellvitge Biomedical Research Unit, Barcelona, Spain; k Infant Jesus Children´s Hospital and Biomedical Research Networking Center on Rare Diseases (CIBERER), ISCIII, Madrid, Spain; l Department of Neurology, Leukodystrophy Clinic, University of Leipzig Medical Center, Leipzig, Germany; m Leukodystrophy Center, Division of Child Neurology, Department of Pediatrics, Weill Cornell Medical College, NewYork-Presbyterian Hospital, New York, NY, USA; n Kremlin-Bicêtre-Hospital, University Paris-Saclay, Paris, France; o Department of Pediatrics, University Medical Center Hamburg Eppendorf, Hamburg, Germany; p Department of Neurology, Harvard Medical School, Massachusetts General Hospital, Boston, MA, USA; q Pediatric Blood and Marrow Transplant Program, Global Pediatrics, Division of Pediatric Blood and Marrow Transplantation, MCRB, University of Minnesota, Minneapolis, MN, USA; r Department of Pediatric Oncology, Hematology and Hemostaseology, University Hospital Leipzig, Leipzig, Germany; s Biomedical Research Networking Center on Rare Diseases (CIBERER), ISCIII, Madrid, Spain

**Keywords:** X-ALD, Neurodegeneration, Biomarker, Neurofilament light chain, Cytokines, GFAP

## Abstract

**Background:**

X-linked adrenoleukodystrophy (X-ALD) is highly variable, ranging from slowly progressive adrenomyeloneuropathy to severe brain demyelination and inflammation (cerebral ALD, CALD) affecting males with childhood peak onset. Risk models integrating blood-based biomarkers to indicate CALD onset, enabling timely interventions, are lacking. Therefore, we evaluated the prognostic value of blood biomarkers in addition to current neuroimaging predictors for early detection of CALD.

**Methods:**

We measured blood biomarkers in a retrospective, male CALD risk-assessment cohort consisting of 134 X-ALD patients and 66 controls and in a phenotype-blinded validation set (25 X-ALD boys, 4–13 years) using Simoa®and Luminex® technologies.

**Findings:**

Among 25 biomarkers indicating axonal damage, astrocye/microglia activation, or immune-cell recruitment, neurofilament light chain (NfL) had the highest prognostic value for early indication of childhood/adolescent CALD. A plasma NfL cut-off level of 8.33 pg/mL, determined in the assessment cohort, correctly discriminated CALD with an accuracy of 96% [95% CI: 80–100] in the validation group. Multivariable logistic regression models revealed that combining NfL with GFAP or cytokines/chemokines (IL-15, IL-12p40, CXCL8, CCL11, CCL22, and IL-4) that were significantly elevated in CALD *vs* healthy controls had no additional benefit for detecting neuroinflammation. Some cytokines/chemokines were elevated only in childhood/adolescent CALD and already upregulated in asymptomatic X-ALD children (IL-15, IL-12p40, and CCL7). In adults, NfL levels distinguished CALD but were lower than in childhood/adolescent CALD patients with similar (MRI) lesion severity. Blood GFAP did not differentiate CALD from non-inflammatory X-ALD.

**Interpretation:**

Biomarker-based risk prediction with a plasma NfL cut-off value of 8.33 pg/mL, determined by ROC analysis, indicates CALD onset with high sensitivity and specificity in childhood X-ALD patients. A specific pro-inflammatory cytokine/chemokine profile in asymptomatic X-ALD boys may indicate a primed, immanent inflammatory state aligning with peak onset of CALD. Age-related differences in biomarker levels in adult *vs* childhood CALD patients warrants caution in predicting onset and progression of CALD in adults. Further evaluations are needed to assess clinical utility of the NfL cut-off for risk prognosis of CALD onset.

**Funding:**

10.13039/501100002428Austrian Science Fund, 10.13039/501100008731European Leukodystrophy Association.

**Research in context**

**Evidence before this study**

We searched PubMed from database inception to January 16th, 2023 for studies on blood biomarkers in X-linked adrenoleukodystrophy (X-ALD) aimed at early detection of onset of cerebral ALD (CALD) using the following terms: “cerebral adrenoleukodystrophy” AND “blood biomarker” OR “neurofilament light” OR “GFAP” OR “chemokines” OR “cytokines”. Many previous studies have reported elevated levels of such biomarkers in the blood of X-ALD patients in general. Ten studies dealt explicitly with biomarkers in the context of CALD; however, we found no study that investigated a blood biomarker-based risk prediction for early-stage cerebral involvement in X-ALD patients.

**Added value of this study**

This multi-centre cohort study shows that among 25 candidate blood biomarkers reflecting axonal damage, astrocyte/microglia activation, and immune cell recruitment, NfL has the highest prognostic value for early detection of progressing brain lesions in X-ALD patients independent of clinical neuroimaging tools. A plasma cut-off value of 8.33 pg/mL, determined in a discovery cohort and applicable to paediatric X-ALD patients 4–18 years, enabled correct classification of the CALD-indicative lesion status in 96% [95% CI: 80–100] of cases within the validation cohort. Our study additionally provides information on a previously unrecognized pro-inflammatory transient cytokine/chemokine profile in asymptomatic X-ALD children lacking cerebral involvement that disappeared in adulthood, potentially indicating a primed immune state aligned with peak age for onset of CALD in childhood. In adult CALD patients, we report increased blood biomarker levels when compared with healthy controls but to a lesser extent than in paediatric CALD patients, despite similar MRI-scored lesion severity. Routine blood sampling for plasma NfL analysis would allow more frequent and less burdensome assessment of brain integrity than with MRI. This would be especially valuable for asymptomatic boys with X-ALD, because the narrow time-window between unnoticed early-stage CALD and ensuing, rapidly progressive and irreversible neurologic decline necessitates prompt treatment for a successful outcome.

**Implications of all the available evidence**

Our study has immediate implications for clinical practice, as it shows that blood-based biomarker risk prediction integrating a defined plasma NfL cut-off could be a useful support tool to stratify paediatric X-ALD patients by their risk for cerebral involvement, thus enabling timely MRI scans and lifesaving interventions by haematopoietic stem cell transplantation or gene therapy.

## Introduction

X-linked adrenoleukodystrohpy (X-ALD, OMIM #300100) is the most common monogenetic disorder of the brain white matter.^1^ X-ALD is caused by mutations in the *ABCD1* gene encoding the ABC-transporter ABCD1 (ALDP), which imports very long-chain fatty acids (VLCFAs) into peroxisomes for degradation.^2^ In ABCD1 deficiency, VLCFAs accumulate in tissues and body fluids, which results in pro-inflammatory skewing of innate immune cells.^3^^,^^4^ Clinically, X-ALD presents with striking phenotypic heterogeneity. The core syndrome, slowly progressive myeloneuropathy (adrenomyeloneuropathy, AMN), typically starts in adulthood and involves spinal cord and peripheral nerves. With peak onset in childhood, male X-ALD patients often experience rapidly progressive cerebral inflammation, demyelination, and axonal degeneration (cerebral ALD, CALD).^1^^,^^5^ What triggers CALD remains unclear; among others, traumatic brain injury^6^ and viral infection have been proposed as causative agents.^7^ Neuroinflammation in CALD is characterized by breakdown of the blood–brain barrier (BBB), infiltration of monocytes/macrophages, and, to a lower extent, T cells.^3^^,^^8^ Hypertrophic astrocytes and activated microglial cells beyond the leading edge of brain lesions indicate both astrogliosis and microgliosis as early events, probably preceding demyelination and invasion of peripheral immune cells.^9^, ^10^, ^11^

At early stages of neuroinflammation, ideally when patients are clinically still presymptomatic, haematopoietic stem cell transplantation (HSCT) or gene therapy can halt the progression of actively demyelinating brain lesions in CALD patients, possibly by replacing dysfunctional and lost microglial cells.^12^, ^13^, ^14^, ^15^ Currently, MRI is the only diagnostic tool to determine CALD onset and eligibility of patients for HSCT or gene therapy. It allows for detection of BBB disruption by visualizing extravasation of gadolinium-based contrast agents into cerebral tissue.^16^

With X-ALD added to newborn screening in many states of the U.S.A and in The Netherlands,^17^^,^^18^ more patients with a life-long risk for CALD conversion will be identified. To detect the onset and progression of CALD, asymptomatic boys undergo an arduous MRI screening programme, often requiring anaesthesia and repeated gadolinium administration.^19^^,^^20^ Thus, there is an urgent need for circulating biomarkers that would provide objective and accurate information on the onset of cerebral involvement in X-ALD patients to 1) supplement and partially replace MRI surveillance, and 2) support early clinical decision making concerning likely progression trajectory and outcome of HSCT/gene therapy.

We recently identified neurofilament light chain (NfL), a cytoskeletal protein reflecting axonal integrity,^21^ as a blood biomarker indicative of inflammatory activity and progression in CALD patients.^5^ NfL is an intermediate filament protein of about 60 kDa that is predominantly found in neurons, specifically in their axons, where it provides structural support to these cells.^21^ When neurons are damaged or undergo degeneration, NfL is released into the CSF and further drained into the blood where it can be reliably measured using the ultrasensitive Single Molecule Array (Simoa®) technology. Crucially, the levels of NfL in the bloodstream were reported to remain unaffected by BBB permeability, which is of utmost importance when utilizing a brain-specific biomarker for early and precise diagnosis.^22^

However, due to the limited number of early-onset CALD participants, our previous pilot study^5^ could not determine the prognostic value of blood NfL for indicating CALD in its earliest stages in individual patients independent of clinical neuroimaging-based prediction. Further, it was unknown whether combining NfL with other circulating biomarkers related to early CALD brain pathology, such as astrocyte/microglia activation and immune cell recruitment, would augment biomarker-aided early detection of CALD. In a scenario of future care, blood biomarkers, once established as indicative of the onset of CALD, could be monitored longitudinally at tight intervals during periods of vulnerability. Alterations implying possible onset of CALD would then prompt referral for MRI.

This study aimed to establish an accurate exclusion of CALD onset based on routine blood sampling and biomarker measurements. Accordingly, we evaluated: (a) whether NfL levels discriminate early cerebral involvement in X-ALD patients, (b) the value of biomarkers indicative of astrocyte/microglia activation or immune cell recruitment to complement NfL for disease onset and staging, and (c) the prognostic significance of these biomarkers to differentiate CALD-indicative demyelination in an independent phenotype-blinded X-ALD validation cohort.

## Methods

### Ethics

The study was approved by the Ethical Review Board of the Medical University of Vienna (EK1613/2019) and informed consent was obtained from participants or legal guardians.

### Study design and participants

Given the X-linked inheritance pattern of X-ALD and the extremely rare occurrence of cerebral ALD in female carriers, this retrospective multicentre cohort study included only male X-ALD patients that were diagnosed based on elevated plasma VLCFAs by gas chromatography-mass spectrometry. The asymptomatic X-ALD and AMN groups included participants without or with myelopathy, respectively, according to Expanded Disability Status Scale (EDSS) scoring but without active inflammatory brain lesions on MRI at blood sampling. Based on MRI, we defined CALD (childhood, CCALD; adult, ACALD) as presence of demyelinating lesions with gadolinium-enhancement or displaying progression (CCALD only); patients with a Loes score ≤2.5 for CALD brain lesion severity were defined as early-stage CALD patients. The Loes grading system ranges from 0 to 34 points based on both location and extent of cerebral demyelination and atrophy in the brain on MRI.^23^ CALD patients who had undergone HSCT or gene therapy were not included in the study. Separate healthy control groups consisted of adult and childhood/adolescent males. Validation was performed on an independent, phenotype-blinded childhood/adolescent X-ALD cohort. Baseline characteristics of patients and controls are shown in Table 1.

**Table 1:** Baseline characteristics of X-ALD variants and healthy controls.

Childhood/adolescent	X-ALD CCALD	X-ALD Asympt	Control	Validation set^c^
Participants, *n*	41^a^	20	17	25
Samples, *n*	45	40	17	25
Plasma, *n*	36	40	10	25
Serum, *n*	9	–	7	–
Age, years	9 (7–12)	9 (6–12)	12 (10–14)	6 (5–8)
MRI severity Loes score	8.5 (2.8–11)	–	N.A.	3.5 (1.1–11.4)

Adult	**X-ALD ACALD**	**X-ALD AMN**	**Control**
Participants, *n*	22^b^	58	49
Samples, *n*	25	84	49
Plasma, *n*	15	66	26
Serum, *n*	10	18	23
Age, years	37 (28–51)	39 (29–45)	39 (31–52)
MRI severity Loes score	8.5 (3.8–10)	–	N.A.

Continuous variables are median (IQR). CCALD (cerebral ALD), childhood/adolescent onset (age ≤21 years).

a This group includes two patients from the asymptomatic X-ALD group who converted to CCALD during the study; ACALD, adult onset (age >21 years).

b This group includes five patients from the AMN set who converted to CALD during the study. Asympt. X-ALD (asymptomatic X-ALD), non-inflammatory childhood/adolescent X-ALD patients before signs of AMN. AMN (adrenomyeloneuropathy), non-inflammatory adult X-ALD patients with myelopathy.

c Validation set, independent cohort of phenotype-blinded childhood X-ALD patients.

### Procedures

Blood samples from X-ALD patients and healthy controls were collected at nine X-ALD research sites (University of Leipzig Medical Center, Germany; Bellvitge Biomedical Research Institute, Spain; Amsterdam University Medical Center, the Netherlands; Kremlin-Bicetre Hospital, France; Medical University of Vienna, Austria; Massachusetts General Hospital, USA; New York-Presbyterian Hospital, USA; University Medical Center Göttingen, Germany; and University of Minnesota, USA) into standard polypropylene EDTA collection tubes and processed at room temperature within 2 h. For serum, tubes were placed upside down at room temperature for 30 min to allow clotting before centrifugation at 2000×*g* for 15 min at room temperature. For plasma, the blood samples were promptly centrifuged at 3500×*g* for 15 min. Serum and plasma samples were aliquoted, pseudonymized and stored at −80 °C.

NfL, GFAP and GM-CSF levels were assessed for blinded samples using Simoa® NF-light Advantage Kit (#103400, Quanterix), GFAP Simoa® Discovery Kit (#102336, Quanterix), and Simoa GM-CSF 2.0 Kit (#102329, Quanterix) on an SR-X Analyzer (Quanterix). The cytokines: IFNγ, IL-1β, IL-4, IL-5, IL-6, CXCL8, IL-10, IL-12p70, IL-22, and TNFα were measured using the Simoa CorPlex™ Human Cytokine 10-plex Panel 1 assay (#85-0329, Quanterix) on an SP-X imaging and analysis system (Quanterix). Briefly, plasma/serum samples were thawed, vortexed and centrifuged at 10,000×*g* for 5 min at room temperature. After diluting samples 4-fold (SR-X analysis) or 1:4.75 (SP-X analysis) in sample diluent buffer, assays were performed following the manufacturer's protocols. Intra-assay variability was evaluated with four blood samples during each run. Intra- and inter-assay coefficients of variation were determined to be <20%. The cytokines/chemokines: CCL11 (eotaxin), CX3CL1 (fractalkine), CXCL1 (GRO), CCL7 (MCP-3), IL-12p40, CCL22 (MDC), IL-15, CXCL8 (IL-8), CXCL10 (IP-10), CCL2 (MCP-1), CCL3 (MIP-1a), CCL4 (MIP-1b), and VEGF-A were measured in blinded plasma/serum samples using Luminex® technology and the MILLIPLEX MAP Human Cytokine/Chemokine Magnetic Bead Panel (HCYTOMAG-60K-06.Hum, Merck) according to the manufacturer's instruction. To assess equivalent measurements for the two types of sample specimens, we additionally performed Simoa® and Luminex® assays on samples derived from a limited number of patients (*n* = 10) where both plasma and serum could be collected simultaneously.

### Statistics

Given the rarity of X-ALD, we decided to use both available plasma and serum as sample types to obtain a sample set of reasonable size. This decision was also based on prior studies that revealed a strong association between plasma and serum NfL measurements, with serum levels showing slightly but not statistically significantly elevated results in univariable analysis.^5^^,^^24^

Continuous variables were described as median and interquartile range (IQR); categorical variables as count and percentage of categories. NfL and GFAP as well as CCL2, CCL22, CXCL1, CXCL10, and CCL3 were log-transformed in all analyses to stabilize residual distributions and reduce the effect of extreme values. IFNγ, IL-1β, IL-4, IL-5, IL-6, CXCL8, IL-10, IL-12p70, IL-22, and TNFα, all measured by Simoa® multiplex analysis and VEGF-A were doubly log-transformed for the same reason, CX3CL1 was square-root-transformed. The values of each marker were compared between conditions using a linear mixed model with condition and an indicator for serum *vs* plasma as fixed factors and patient ID as random factor to account for multiple measurements per patient. For NfL and GFAP, also a fixed factor indicating the site of biomarker measurement was added. Differences between conditions are reported as least squares means from these models. In case of NfL and GFAP, the mean difference was back-transformed from the log-scale, resulting in a geometric mean ratio. Reported *p*-values of pre-selected contrasts between conditions were adjusted for multiple testing using the method of Bonferroni-Holm. Confidence intervals for differences or mean ratios are adjusted using the method of Bonferroni.

Conditional model residual distributions were checked for outliers and potentially influential observations. Suspicious observations were identified for IL-15, CCL11, TNFα, CCL2, CXCL10, and CCL3. Sensitivity analyses were performed by repeating the linear mixed model without these observations. Only for CCL3, results of the sensitivity analysis were reported in addition to original results, since a relevant change of effect estimate or *p*-value was observed.

Each marker's association with Loes score was quantified using Spearman's correlation coefficient partialized for the indicator of serum *vs* plasma. For graphical representation of this association, a linear regression of log (NfL) on a linear and a quadratic term for Loes score was fitted in GraphPad 7.00. The ability of each marker to discriminate between inflammatory CALD and non-inflammatory X-ALD was investigated by a univariable logistic regression model. Sensitivity and specificity of varying cut-offs and the resulting area under the receiver operating characteristics (ROC) curve (AUC) were obtained from these models. For obtaining a bias corrected and accelerated (BCa) percentile confidence interval for the AUC, 1000 bootstrap samples were drawn, blocked on patient ID to account for the clustering of measurements.^25^ A *p*-value of the marker's effect was obtained by the same bootstrap method. For NfL in children, a cut-off was chosen such that maximum specificity was obtained while keeping sensitivity at 100%. Accuracy (percentage of correctly identified patients) for the validation cohort is reported with 95% confidence interval. Multivariable logistic regression models were used to investigate the gain in AUC when adding log (GFAP) to log (NfL) as predictors, and a potentially different effect of NfL in children and adults (by adding an interaction term). All statistical analyses were performed using SAS version 9.4 (SAS Institute Inc., 2016). All reported *p*-values are two-sided. *p*-values below 0.05 were regarded to indicate statistical significance. All graphs were produced using GraphPad Prism 7.00.

### Role of the funders

The funders of the study had no role in study design, data collection, data analysis, data interpretation, or writing of the report.

## Results

### Study design

For the discovery study, in total, 260 plasma or serum samples were obtained from 200 participants (134 X-ALD patients and 66 healthy volunteers) from eight X-ALD centres (median number of measurements per patient: 1; maximum: 6). The cohort consisted of: CCALD (*n* = 41), ACALD (*n* = 22), asymptomatic X-ALD (*n* = 20), and adult non-inflammatory AMN (*n* = 58) patients and healthy controls (children/adolescents, *n* = 17; adults, *n* = 49). Given the rarity of X-ALD, we used both available plasma and serum to obtain a sample set of reasonable size and incorporated a sample type indicator for adjustment in all statistical analyses. An independent validation set (provided by T. Lund, University of Minnesota, USA) comprised single plasma samples from 25 phenotype-blinded, male childhood/adolescent X-ALD patients. Baseline characteristics of the participants are shown in Table 1 and more details for the CALD patients and the validation cohort in Supplementary Tables S1 and S2

### NfL discriminates early-stage CALD from asymptomatic X-ALD in childhood/adolescence but not in adulthood

**Fig. 1:**
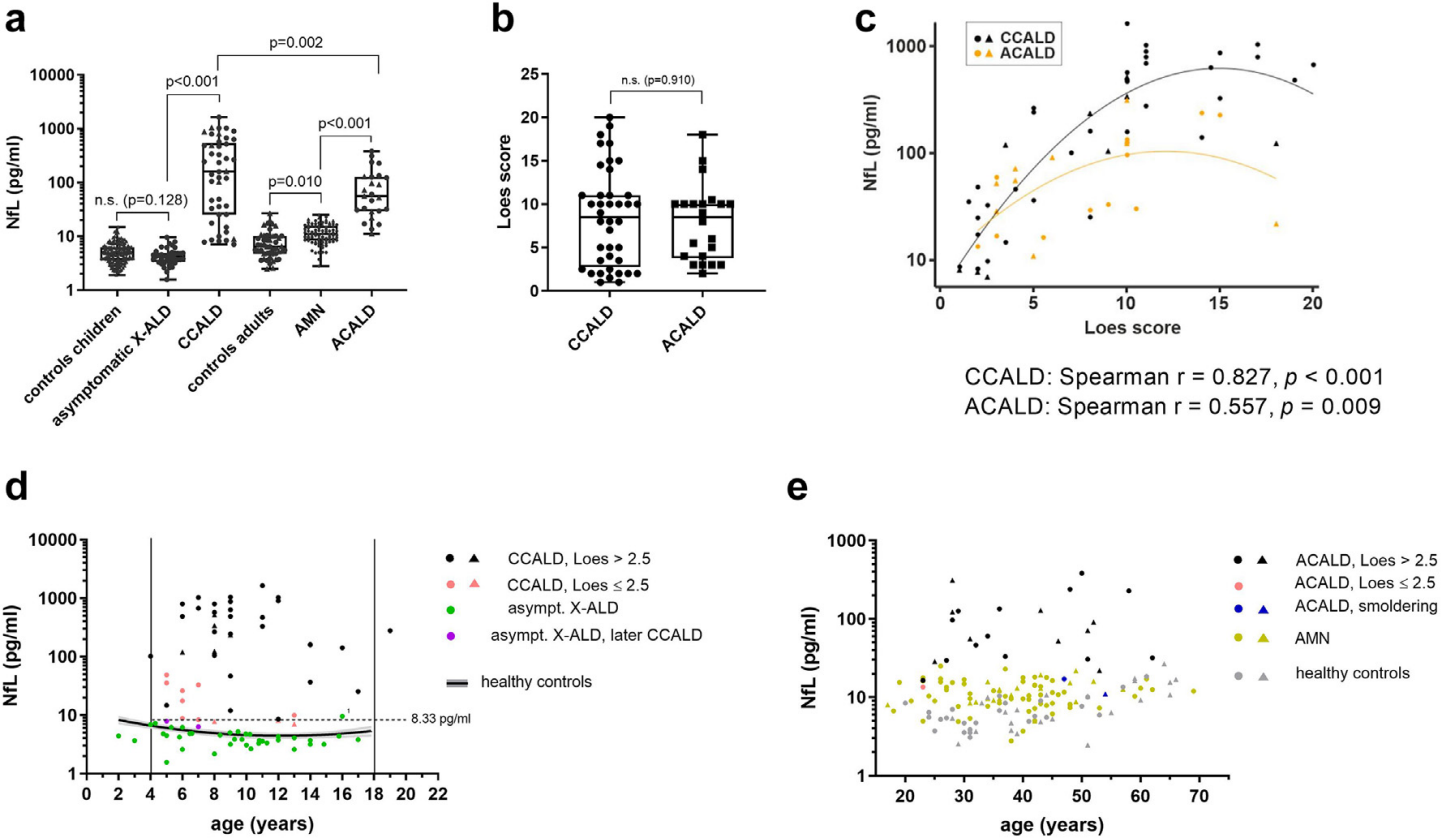
**NfL has a higher capacity to discriminate onset of CALD in children than in adults with X-ALD. (a)** NfL in plasma and serum samples of childhood/adolescent X-ALD patients (asymptomatic X-ALD, *n* = 20, median age = 9.4 years, total sample number = 40; inflammatory CALD [CCALD], *n* = 41, median age = 9 years, total sample number = 45), adult X-ALD patients (non-inflammatory AMN, *n* = 58, median age = 39 years, total sample number = 84; adult CALD [ACALD], *n* = 22, median age = 37 years, total sample number = 25) and healthy controls (childhood/adolescents: *n* = 15, with additional NfL levels of 138 boys measured using the same methodology and described in,^26^ median age = 11 years, total sample number = 153; adults: *n* = 49, median age = 39 years, total sample number = 49). The data are depicted as boxplots (median ± IQR). Total sample numbers include samples collected longitudinally for some patients during disease progression. Comparison of log (NfL) levels was done using a linear mixed model adjusted for sample type (serum *vs* plasma) with the addition of a random ID factor to account for the longitudinal sampling. Multiple testing was corrected by the Bonferroni-Holm method. Adjustment of the group comparisons for age differences did not change the significance or the reported *p*-values. **(b)** Comparison of MRI severity (Loes score, indicating location and activity of the inflammatory myelin destruction) in groups of CCALD (*n* = 38, median age = 9 years, total sample number = 40) and adult ACALD (*n* = 19, median age = 37 years, total sample number = 22) patients. The data are depicted as boxplots (median ± IQR). **(c)** Association between plasma/serum NfL levels and Loes scores in CCALD (*n* = 38, median age = 6 years, total sample number = 42, black symbols) and ACALD patients (*n* = 22, median age = 37 years, total sample number = 24, orange symbols). The Linear mixed model included a fixed factor for sample type and a random ID factor as well as a linear and quadratic term for the Loes score. **(d**–**e)** Association of NfL and age in samples from **(d)** CCALD (Loes >2.5, black symbols; early onset CCALD, Loes ≤2.5, pink symbols) and asymptomatic X-ALD patients (asympt. X-ALD, dark green symbols; patients who later converted to CALD, lilac symbols). For curve fitting using linear regression including a quadratic term of log (NfL) on age in healthy control children/adolescents, data described by^26^ was used. The cut-off value of 8.33 pg/mL to discriminate plasma samples of CCALD from asymtomatic X-ALD children is indicated by a dotted line. ^1^Asymptomatic X-ALD patient 1 (asympt. X-ALD 1). **(e)** Association of NfL and age in samples from ACALD (Loes >2.5, black symbols; early onset ACALD, Loes ≤2.5, pink symbols; smouldering ACALD, blue symbols), non-inflammatory AMN, light green symbols, and adult healthy controls (grey symbols). Serum samples are indicated by triangles and plasma samples by circles.

With acute neuroinflammatory demyelination in CCALD or ACALD, blood NfL concentrations were markedly increased when compared with asymptomatic childhood/adolescent X-ALD or non-inflammatory AMN patients of similar age, respectively (raw data children/adolescents: median 158.8; IQR [25.3–545.1] pg/mL *vs* 4.2 [3.3–4.9] pg/mL; model estimate of mean ratio CCALD *vs* asymptomatic X-ALD: 24.2, CI: 13.1–44.6; adj. *p* < 0.001, linear mixed model; raw data adults: 55.8 [29.1–127.6] pg/mL *vs* 11.0 [8.3–15.7] pg/mL; model estimate of mean ratio ACALD *vs* AMN: 4.3, CI: 2.5–7.5; adj. *p* < 0.001, linear mixed model; Fig. 1a) confirming our previous results.^5^ In contrast to adult AMN patients with ongoing myelopathy and progressive axonal damage, blood NfL levels in yet asymptomatic X-ALD children/adolescents were not significantly different from healthy controls with comparable age distribution (4.2 [3.3–4.9] *vs* 4.8 [3.6–6.3] pg/mL; model estimate of mean ratio: 1.62, CI: 0.71–3.66; adj. *p* = 0.128, linear mixed model). Direct comparison between the CCALD and ACALD groups revealed significantly lower blood NfL levels in ACALD patients (158.8 [25.3–545.1] *vs* 55.8 [29.1–127.6] pg/mL; model estimate of mean ratio: 0.5, CI: 0.3–0.8; adj. *p* = 0.002, linear mixed model; Fig. 1a), despite similar distribution (*p* = 0.910, Wilcoxon's rank-sum test, Fig. 1b) of MRI Loes-scored myelin abnormalities. This relationship between NfL and MRI brain lesion severity was also reflected in linear regression analysis, with CCALD patients showing a stronger, statistically significant dependence of log (NfL) on the status of white matter abnormalities (*r* = 0.827, *p* < 0.001, Spearman's correlation) than ACALD patients (*r* = 0.557, *p* = 0.009, Spearman's correlation, Fig. 1c).

Because the increase in NfL coincided with early signs of CALD onset, we compared blood NfL levels of early CCALD patients (Loes score ≤2.5) with those of asymptomatic X-ALD of similar age (Fig. 1d). In all of the tested patients with early CCALD (*n* = 10), the NfL levels exceeded those of asymptomatic X-ALD children (*n* = 20, total sample number = 40) except one (asympt. X-ALD 1, age 16 years; Fig. 1d). For this patient with modestly elevated NfL levels (9.62 pg/mL), compared with healthy controls of similar age, MRI analysis excluded presence of CALD; however, reassessment of clinical data revealed an acute adrenal crisis with psychiatric abnormalities. In adult X-ALD, NfL levels did not separate CALD from non-inflammatory (AMN) patients as effectively as in childhood/adolescent X-ALD (Fig. 1e). In 5/22 ACALD patients, we observed blood NfL values within the range of AMN patients (Fig. 1e). Longitudinal sampling of two patients before and after conversion to CCALD and of six asymptomatic X-ALD children is shown in Supplementary Figure S1.

Using logistic regression analysis, we evaluated the diagnostic performance of blood NfL for discriminating between asymptomatic X-ALD and CCALD conditions in children/adolescents older than 4 years. We chose this age because CALD most frequently starts after the age of 4 in childhood and, furthermore, in children NfL is not linearly associated with age, necessitating a reasonably sized group of asymptomatic X-ALD of similar age for comparison. With the majority of blood samples in our study being plasma (CCALD: 82%, asymptomatic X-ALD: 100%), here we focused on plasma as sample type. Statistical analysis confirmed that blood NfL has a significant effect on the indicator CCALD *vs* asymptomatic (McFadden's R^2^ = 0.902, *p* < 0.001, logistic regression). In ROC curve analysis, an AUC of 0.997 (95% CI: 0.983–1.00; Supplementary Figure S2) identifies NfL as an excellent marker to differentiate participants with or without cerebral involvement. As a cut-off value for NfL, we propose the value that still gives 100% sensitivity with maximum specificity. In our experiments, the plasma cut-off value of 8.33 pg/mL distinguished asymptomatic probands from CALD children/adolescents with a sensitivity of 100% and a specificity of 95.2% (Fig. 1d, Supplementary Figure S2). In adult X-ALD patients, logistic regression analysis of plasma samples from ACALD *vs* AMN patients revealed an AUC of 0.955 (95% CI: 0.876–1.000, Supplementary Figure S2), indicating NfL as a significant indicator for CALD onset also in adulthood.

### Increased blood GFAP indicative of astrocyte damage is associated with neuroinflammation only in childhood/adolescent CALD

**Fig. 2:**
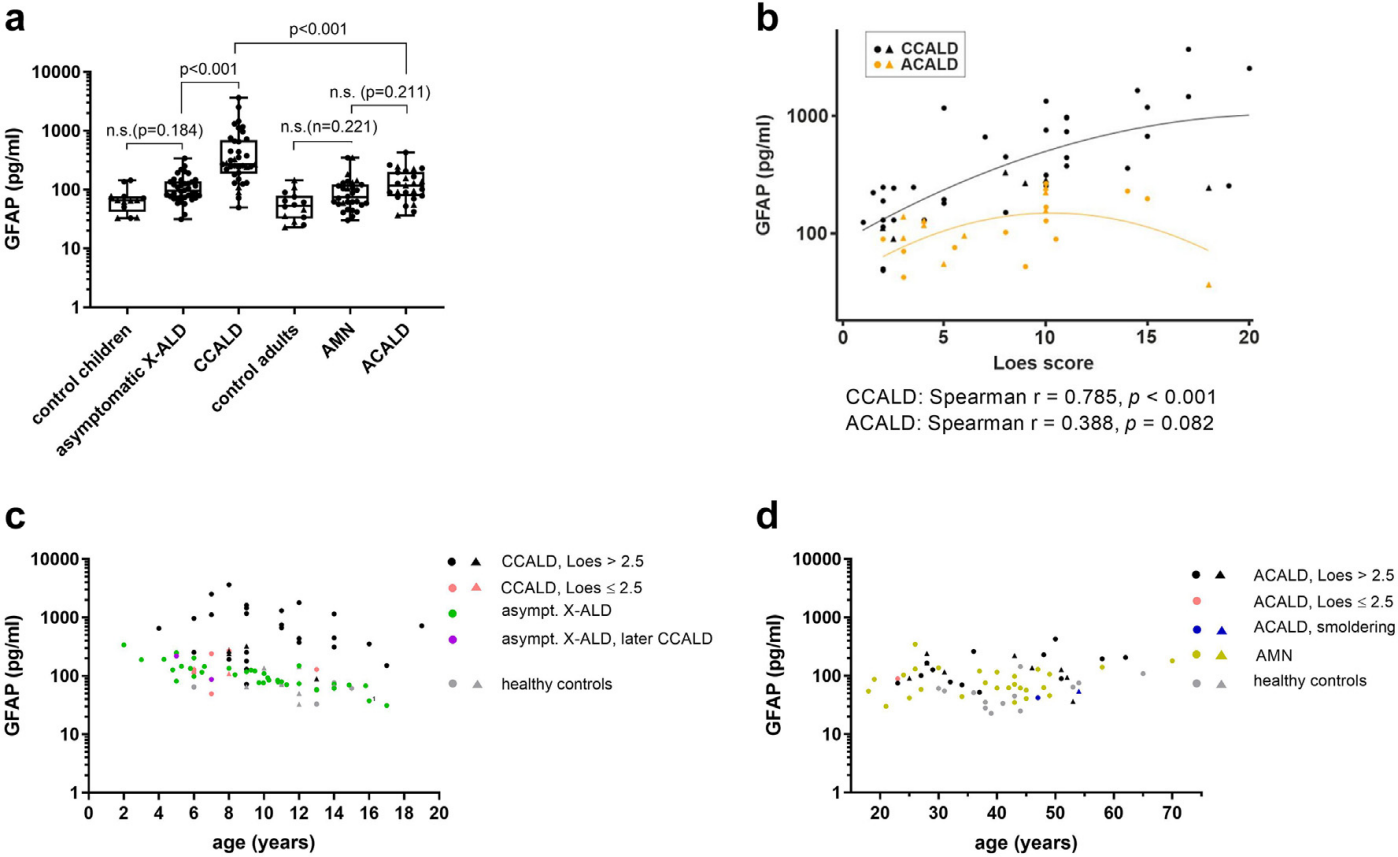
**Blood GFAP indicative of astrocyte damage is associated with CALD only in childhood/adolescent X-ALD. (a)** GFAP levels in samples of childhood/adolescent X-ALD patients (asymptomatic X-ALD, *n* = 20, median age = 9.4 years, total sample number = 40; CCALD, *n* = 38, median age = 9 years, total sample number = 42), adult X-ALD patients (non-inflammatory AMN, *n* = 23, median age = 40 years, total sample number = 27; ACALD, *n* = 22, median age = 37 years, total sample number = 25), and healthy controls (childhood/adolescents: *n* = 15, median age = 12 years, total sample number = 15; adults: *n* = 14, median age = 42 years, total sample number = 14). The data are depicted as boxplots (median ± IQR). Total sample numbers include samples collected longitudinally from some patients during disease progression. Comparison of log (GFAP) levels was done using a linear mixed model adjusted for sample type (serum *vs* plasma) with the addition of a random ID factor to account for the longitudinal sampling. Multiple testing was corrected by the Bonferroni-Holm method. Adjustment of the group comparisons for age differences did not change the significance or the reported *p*-values. **(b)** Association between plasma/serum GFAP levels and brain lesion severity (Loes score) in CCALD (*n* = 38, median age = 6 years, total sample number = 42, black symbols) and ACALD patients (*n* = 22, median age = 37 years, total sample number = 24, orange symbols). The Linear mixed model included a fixed factor for sample type and a random ID factor as well as a linear and quadratic term for the Loes score. **(c**–**d)** Association of GFAP and age in samples from **(c)** CCALD (Loes >2.5, black symbols; early onset CALD, Loes ≤2.5, pink symbols) and asymptomatic X-ALD patients (asympt. X-ALD, dark green symbols; patients who later converted to CALD, lilac symbols; ^1^Asymptomatic X-ALD patient 1, asympt. X-ALD 1) and **(d)** ACALD (Loes >2.5, black symbols; early stage ACALD, Loes ≤2.5, pink symbols; smouldering ACALD, blue symbols), non-inflammatory AMN (light green symbols), and adult healthy controls (grey symbols). Serum samples are indicated by triangles and plasma samples by circles (a–d).

Astrocytes are major constituents of the BBB, and both BBB damage and astrogliosis are prominent features of CALD pathology.^9^^,^^27^^,^^28^ Thus, we next assessed whether release of the intermediate filament protein glial fibrillary acidic protein (GFAP) into the blood of X-ALD patients is increased upon conversion to CALD. Before onset of neuroinflammation, median GFAP levels in both asymptomatic X-ALD children/adolescents and AMN patients with progressive myeloneuropathy were higher than in controls of similar age (children/adolescents: median 95.4 [73.3–137.8] *vs* 65.3 [41.8–76.5] pg/mL; adults: 73.8 [56.2–122.7] *vs* 53.2 [32.4–78.9] pg/mL; Fig. 2a). However, this trend towards increased blood GFAP did not reach statistical significance for either asymptomatic X-ALD (model estimate of mean ratio: 1.6, CI: 0.8–3.2; *p* = 0.184, linear mixed model) or for AMN patients (model estimate of mean ratio: 1.4, CI: 0.8–2.4; *p* = 0.221). With conversion to inflammatory CALD, GFAP levels were markedly increased in CCALD (270.5 [186.3–696.9] pg/mL; model estimate of mean ratio: 3.4, CI: 2.1–5.5; adj. *p* < 0.001, linear mixed model) but not in ACALD patients (117.2 [77.0–200.7] pg/mL; model estimate of mean ratio: 1.4, CI: 0.8–2.3; adj. *p* = 0.221, linear mixed model; Fig. 2a). Longitudinal measurements of GFAP in asymptomatic X-ALD children and CCALD patients before and after conversion are shown in Supplementary Figure S3. The amount of GFAP in the blood reflected the brain lesion load in CCALD as measured by the MRI severity score (*r* = 0.785, *p* < 0.001, Spearman's correlation), whereas in ACALD patients the weak association between blood GFAP and Loes score did not reach statistical significance (*r* = 0.388, *p* = 0.082, Spearman's correlation; Fig. 2b). However, when plotted against age, neither in paediatric nor adult X-ALD patients, did blood GFAP levels efficiently separate CALD from asymptomatic X-ALD or AMN (Fig. 2c and d).

Consistently, logistic regression for assessing the utility of blood GFAP to discriminate the onset of neuroinflammation in X-ALD patients revealed that GFAP is a better predictor for CALD in childhood/adolescence (AUC 0.881, 95% CI 0.757–0.968) than in adulthood (AUC 0.643, 95% CI: 0.503–0.813; Supplementary Figure S2). However, GFAP in addition to NfL did not further improve the ability of NfL to discriminate inflammatory CALD from non-inflammatory X-ALD in paediatric patients (AUC 0.996 *vs* 0.997 for NfL alone).

### Blood cytokine/chemokine levels in asymptomatic X-ALD children/adolescents indicate a primed pro-inflammatory state

**Fig. 3:**
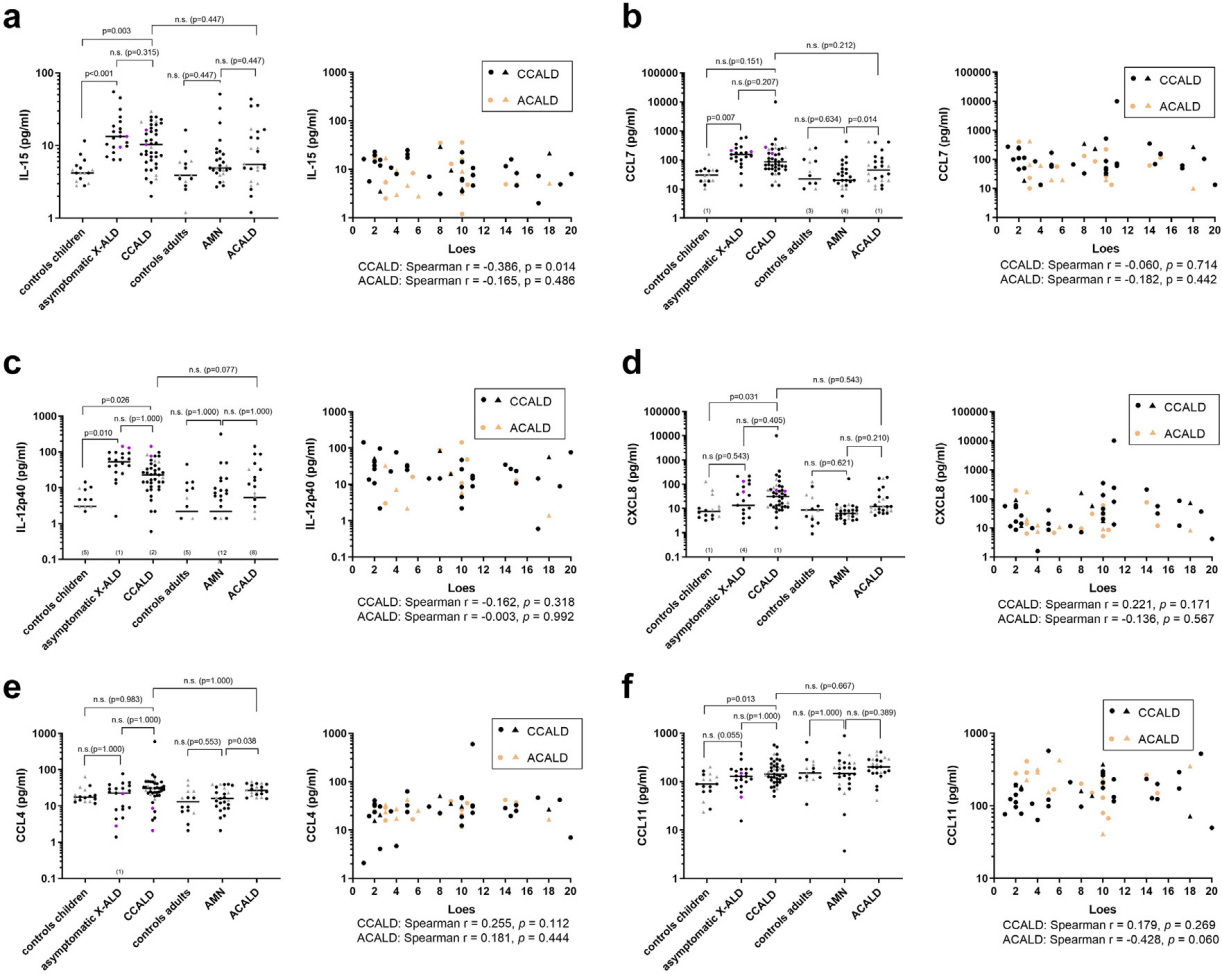
**Increased blood IL-15, CCL7 and IL-12p40 levels indicate an X-ALD immanent pro-inflammatory status in childhood that does not persist in adulthood.** Plasma and serum samples derived from childhood/adolescent X-ALD patients (asymptomatic X-ALD, *n* = 20, median age = 9 years, total sample number = 21; inflammatory CALD, *n* = 37, median age = 9 years, total sample number = 41), adult X-ALD patients (non-inflammatory AMN, *n* = 24, median age = 40 years, total sample number = 27; adult CALD, *n* = 24, median age = 40 years, total sample number = 24), and healthy controls (childhood/adolescents: *n* = 16, median age = 12 years, total sample number = 16; adults: *n* = 14, median age = 42 years, total sample number = 14) were used to determine blood levels of and their association with Loes scores for MRI-severity for **(a)** IL-15, **(b)** CCL7, **(c)** IL-12p40, **(d)** CXCL8, **(e)** CCL4, and **(f)** CCL11 (linear mixed models). The median is indicated by a horizontal line in the dot plots. Total sample numbers include several samples collected longitudinally from the same patients during disease progression. Reported Spearman's *r* have been partialized for the sample type serum or plasma. Serum samples are indicated by grey triangles and plasma samples by black circles. Childhood X-ALD patients before and after conversion to CALD are indicated by lilac circles. The number of data points that were below the detection limit are indicated in brackets on the x-axis above the respective group.

Innate immune cell activation and microglia damage are thought to precede both myelin breakdown and neuronal injury in the brain of CALD patients.^10^^,^^11^ Therefore, we assessed cytokines/chemokines and growth factors indicative of microglia and/or astrocyte activation and immune cell recruitment as blood biomarkers to complement NfL either for detecting early stage CALD or for estimating disease progression. We used multiplex Luminex® bead arrays to measure blood levels of the cytokines/chemokines: CCL11 (eotaxin), CX3CL1 (fractalkine), CXCL1 (GRO), CCL7 (MCP-3), IL-12p40, CCL22 (MDC), IL-15, CXCL8 (IL-8), CXCL10 (IP-10), CCL2 (MCP-1), CCL3 (MIP-1a), CCL4 (MIP-1b), and VEGF-A (Fig. 3; Supplementary Figure S4). In addition, we used Simoa CorPlex™ cytokine panel assay to determine IFN-γ, IL-1b, IL-4, IL-5, IL-6, CXCL8, IL-10, IL-12p70, IL-22, and TNFα (Supplementary Figure S6) and Simoa single assay to measure GM-CSF levels (Supplementary Figure S7). When compared to healthy controls of similar age, onset of neuroinflammation was associated with significantly elevated blood levels of IL-15 (10.3 [6.1–18.35] pg/mL *vs* 4.2 [3.3–5.2] pg/mL, adj. *p* = 0.003), IL-12p40 (22.8 [9.9–44.8] pg/mL *vs* 3 [0–7.9] pg/mL, adj. *p* = 0.026, linear mixed model), CXCL8 (31.4 [11.7–66.1] pg/mL *vs* 7.6 [4.4–11.9] pg/mL, adj. *p* = 0.031, linear mixed model), CCL11 (142.2 [109.5–221.4] pg/mL *vs* 89.4 [62.6–136.4] pg/mL, adj. *p* = 0.013, linear mixed model; Fig. 3), CCL22 (1099 [929.8–1434] pg/mL *vs* 993.5 [763–1324] pg/mL, adj. *p* = 0.041, linear mixed model), and IL-4 (2.0 [1.4–4.9] pg/mL *vs* 1.1 [0.7–2.1] pg/mL, adj. *p* = 0.044, linear mixed model) in childhood/adolescent X-ALD patients (Fig. 3, Supplementary Figure S4, Supplementary Figure S6). Upon correction for potentially influential observations/outliers, sensitivity analysis indicated also a significant increase in blood CCL3 levels in CCALD patients (model estimate of mean ratio: 0.1, CI: 0.03–0.6, Supplementary Figure S4). Of note, in asymptomatic X-ALD children, blood levels of IL-15 (13.3 [9.5–19.9] pg/mL *vs* 4.2 [3.3–5.2] pg/mL, adj. *p* < 0.001, linear mixed model), IL-12p40 (53.6 [25.7–92.8] *vs* 3 [0–7.9] pg/mL, adj. *p* = 0.010, linear mixed model), and CCL7 (156.3 [95.8–234] *vs* 30.15 [18.8–42.4] pg/mL, adj. *p* = 0.007, linear mixed model) were already significantly elevated when compared with healthy controls of similar age (Fig. 3a–c). Longitudinal sampling of CCALD patients and asymptomatic X-ALD children is shown in Supplementary Figure S5. Linear regression analysis to elucidate the relationship of the tested markers with MRI-scored brain lesion severity (Loes score) revealed a statistically significant negative dependence of log (IL-15) on the status of myelin abnormalities in CCALD patients (r = 0.386, *p* = 0.014, Spearman's correlation; Fig. 3a). ROC analysis investigating the capability of these cytokines/chemokines to differentiate cerebral involvement in X-ALD patients showed that none of these biomarkers further improved the ability of NfL to discriminate the onset of CALD in X-ALD children (Supplementary Table S3).

In non-inflammatory AMN patients, neither IL-15, IL12-p40, nor CCL7 were increased compared with healthy controls of similar age (Fig. 3), possibly pointing to a role of an X-ALD immanent, pro-inflammatory primed activation state in asymptomatic X-ALD patients during a critical time window in childhood/adolescence, when peak onset of CALD occurs. When compared with healthy controls, the presence of myelopathy in AMN patients was reflected by significantly altered blood levels of CXCL10 (478.4 [344.7–730.1] pg/mL *vs* 258.8 [186.3–435.9] pg/mL, adj. *p* = 0.042, linear mixed model), CCL22 (740.9 [554.4–941.2] pg/mL *vs* 558.6 [298–900.2] pg/mL, adj. *p* = 0.004, linear mixed model), Supplementary Figure S4b and f; and TNFα (2.0 [1.2–2.2] pg/mL *vs* 2.2 [1.7–3.7] pg/mL, adj. *p* = 0.014, linear mixed model), and IL-12p70 (0.4 [0.4–0.5] *vs* 0.9 [0.5–1.1] pg/mL, adj. *p* = 0.005, linear mixed model), Supplementary Figure S6f and j. After onset of neuroinflammation in adult X-ALD patients, CCL7 (45.0 [19.8–109] pg/mL *vs* 19.8 [10.1–47.9] pg/mL, adj. *p* = 0.014, linear mixed model), CCL4 (26.8 [20.2–38.5] pg/mL *vs* 16.2 [9.3–32.2] pg/mL, adj. *p* = 0.038, linear mixed model, Fig. 3b–e); as well as CXCL1 (1120 [700.1–1627] pg/mL *vs* 713.7 [172.1–1270] pg/mL, adj. *p* = 0.035, linear mixed model, Supplementary Figure S4a) and IL-12p70 (0.9 [0.5–1.6] pg/mL *vs* 0.4 [0.4–0.5] pg/mL, adj. *p* = 0.004; Supplementary Figure S6j) but none of the markers identified in CCALD patients were found to be elevated when compared with AMN patients.

### The high prognostic value of NfL enables exclusion of CALD in children with X-ALD

**Fig. 4:**
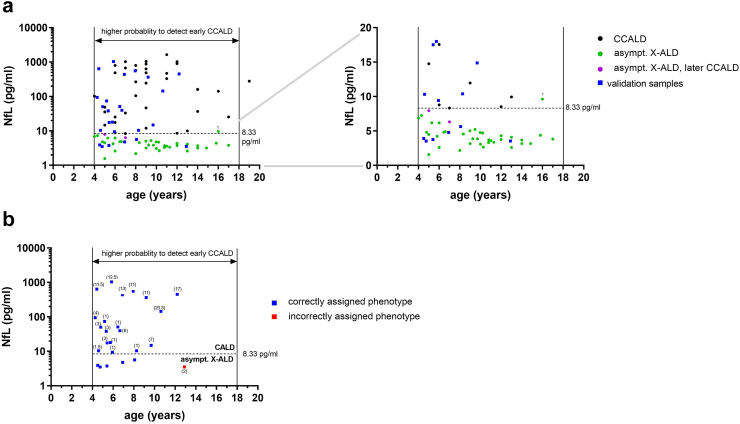
**Evaluation of plasma NfL to indicate cerebral involvement in X-ALD patients independent of clinical neuroimaging-based assessment. (a)** Association of plasma NfL and age in samples from the development cohort of childhood/adolescent X-ALD patients (CALD, *n* = 38, median age = 9 years, total sample number = 40, black symbols; and asymptomatic X-ALD, *n* = 20, median age = 9.4 years, total sample number = 40, green symbols; asymptomatic X-ALD patients who later converted to CALD, lilac symbols) and the phenotype-blinded childhood/adolescent validation cohort (*n* = 25, median age = 6 years, total sample number = 25). **(b)** Differentiation of CALD onset in the validation cohort based on the plasma NfL cut-off level of 8.33 pg/mL. Blue symbols indicate correctly and red incorrectly assigned phenotypes. Numbers in brackets indicate the MRI brain lesion severity (Loes score), disclosed after NfL–based designations.

Among 25 tested candidate blood biomarkers reflecting different aspects of CALD pathobiology such as axonal damage, astrocyte/microglial activation and immune cell recruitment, blood NfL surpassed all other markers regarding the ability to indicate cerebral involvement and inflammatory neurodegeneration in X-ALD patients. Thus, focusing on childhood/adolescent X-ALD patients, we evaluated the prognostic value of blood NfL to evidence CALD-indicative demyelination, without prior knowledge of clinical neuroimaging-based assessment, in a plasma validation set from phenotype-blinded X-ALD patients (*n* = 25; aged 4–13 years, median age = 6 years; details in Supplementary Table S2). Applying the plasma NfL cut-off value of 8.33 pg/mL, we classified the presence or absence of CALD lesions for the respective patients (CALD-indicative demyelination: plasma NfL ≥8.33 pg/mL; asymptomatic X-ALD: plasma NfL <8.33 pg/mL, Fig. 4). Decoding revealed that this NfL cut-off correctly classified 24 out of the 25 boys with X-ALD from the validation cohort according to their MRI-assigned clinical status of cerebral integrity, indicating a prognostic accuracy of 96% [95% CI: 80–100] for blood NfL levels to correctly differentiate CALD in childhood/adolescent X-ALD patients.

Only one X-ALD patient (ID Val25) was assigned to the wrong phenotype group based on NfL (asymptomatic X-ALD), despite MRI-documented CALD onset with Loes score 2 (Fig. 4b). In addition to low NfL compared with other CCALD patients (3.54 pg/mL *vs* median 158.8 [25.26–545.1] pg/mL), CCALD patient ID Val25 (age, 12.9 years) also had a very low plasma GFAP level (24.10 pg/mL) compared with either healthy controls of similar age or other CCALD patients (median 65.3 [41.8–76.5] pg/mL and 95.4 [73.3–137.8], respectively). Of note, elevated plasma NfL ≥8.33 pg/mL also correctly indicated CCALD in two X-ALD patients (ID Val13, plasma NfL 9.42 pg/mL and ID Val20, plasma NfL 10.39 pg/mL) with MRI Loes score 1. Both patients presented with CALD-characteristic brain lesions but did not show extravasation of gadolinium-based contrast agents into cerebral tissue indicating a yet intact BBB.

## Discussion

The neuroinflammatory CCALD variant of X-ALD continues to be among the most severe neurological disorders that affect children. With X-ALD being added to newborn screening programmes in the U.S.A and Europe, the next challenge is the early diagnosis of cerebral involvement in X-ALD patients at risk, as prompt treatment is crucial for favourable outcome and quality of life. The difficulty here is the phenotypic heterogeneity in X-ALD, and to weigh the risk of a fatal disease course against risks associated with the HSCT or gene therapy procedures. Even among children and adolescents who develop CCALD, the time of onset and symptoms vary considerably.

Currently, asymptomatic boys are screened for onset and progression of CALD through a demanding MRI monitoring programme that often involves anaesthesia and multiple gadolinium administrations. In addition to the burden of these procedures, for some families access to this screening may be difficult. Therefore, it is important to identify circulating biomarkers that can provide accurate and objective information on the onset of cerebral involvement in X-ALD patients.^29^ Such biomarkers would be a valuable supplement to the existing MRI surveillance programme and could potentially reduce the number of MR scans needed.

In the present work, we assessed blood biomarker-based risk prediction to discriminate the early onset of neuroinflammation in X-ALD patients. We provide evidence supporting blood NfL levels as the best prognostic liquid biomarker for the presence of CALD-suggestive lesions in the brain of both childhood/adolescent and adult X-ALD patients. Once NfL was included in the model, no other biomarkers improved the discrimination between X-ALD phenotypes in paediatric patients markedly. In our X-ALD validation cohort of 25 childhood/adolescent X-ALD patients (4–13 years), a plasma NfL cut-off value of 8.33 pg/mL detected CALD with a prognostic capacity of 96% [95% CI: 80–100].

Blood NfL is now a well-established prognostic marker indicating disease severity and/or progression across various neurodegenerative diseases including, for example, multiple sclerosis and Alzheimer's disease.^21^^,^^30^^,^^31^ Recently, three independent studies demonstrated that blood NfL accurately reflects disease activity in X-ALD patients.^5^^,^^32^^,^^33^ Currently, measurement of blood NfL has not yet been implemented in the clinical management of X-ALD. However, a special need arises from increasing numbers of patients identified in newborn screening programmes, who are now regularly monitored for CALD-onset by MRI techniques. One obstacle for implementing blood NfL into clinical practice has been the lack of an accurate cut-off level defining pathological elevation and start of cerebral involvement. In this study, we used logistic regression analysis and blood samples from a discovery cohort of childhood CALD and asymptomatic X-ALD patients to determine a plasma NfL cut-off value of 8.33 pg/mL to differentiate CALD-indicative cerebral lesions with a sensitivity of 100% and a specificity of 95.2% in childhood/adolescent X-ALD patients (4–18 years). Assessment of this cut-off level within an independent validation cohort of phenotype-blinded childhood/adolescent X-ALD patients enabled the correct classification of X-ALD phenotypes in 24 out of 25 X-ALD patients. Despite 96% prognostic accuracy of NfL, the inability to detect the ongoing early brain lesion, which was identified by MRI, in one boy with CALD from the validation cohort warrants future investigations using enlarged patient numbers. Specifically, it needs to be clarified why occasional blood samples from CCALD patients (within our study, 1/61 investigated CCALD cases) may exhibit atypically low NfL levels, despite progressive cerebral damage, and how this correlates with disease progression and treatment outcome.

Adult CALD patients presented with significantly lower blood NfL levels than childhood/adolescent CALD patients. This may challenge the distinction of CALD-indicative disease signals from changes associated with progression of myelopathy in AMN. By consequence, a fixed NfL cut-off could lead to misclassification and, thus, to a significant false negative prognostic rate for CALD in adult X-ALD patients. Further, a subset of ACALD patients present with diffuse, continuously worsening smouldering lesions that are associated with only modestly increased NfL levels.^5^ Thus, smouldering ACALD patients may remain unrecognized by a fixed neuroinflammation-indicative NfL cut-off. Hence, for adult X-ALD patients with CALD onset, the ability of blood NfL level to guide clinical decision making is currently limited. Here, tight longitudinal assessment of individual patients is needed to improve the prognostic value of absolute or relative changes between MRI assessments.

GFAP, another biomarker used as diagnostic instrument in the setting of neuroinflammation and recently FDA-approved for mild traumatic brain injury,^34^ had no added value in our model to separate CALD from asymptomatic X-ALD patients. Although being significantly elevated with onset and progression of cerebral involvement in CCALD patients, GFAP had lower discriminative capability for CALD-indicative demyelination than NfL. Of note, when compared to healthy controls of similar age, median blood GFAP levels were already 30% higher in still asymptomatic childhood/adolescent X-ALD patients. This might point to an X-ALD-immanent increase in GFAP, indicative of astrocyte damage associated with ABCD1 deficiency and concurrent accumulation of VLCFAs in tissues and body fluids independent of phenotype. In our study, AMN was not associated with increased blood GFAP levels compared with healthy controls. These findings contrast with the recent observation by van Ballegoij and colleagues,^33^ who found that myelopathy in AMN patients is significantly linked to elevated GFAP levels. The reason for these conflicting results may lie in differences of AMN group composition, such as severity of myelopathy, age and a smaller sample size.

Our data further indicate upregulation of the cytokines IL-15, IL-12p40, and CCL7 in the blood of yet asymptomatic children with X-ALD at a highly susceptible age for developing CALD. Intriguingly, these cytokines play a role in neuroinflammation and may indicate a primed state for disease onset. IL-15 is a danger signal that communicates the health status of tissues to the immune system.^35^ It promotes activation, proliferation and survival of natural killer and T cells but also stimulates CXCL8 and CCL2 secretion in antigen presenting cells including monocytes/macrophages. Under conditions of neuroinflammation, IL-15 is mainly secreted by astrocytes but can also be detected on microglia surrounding infiltrating CD8+ T cells in multiple sclerosis lesions.^36^ Of note, downstream signalling of IL-15 cumulates in activation of the stress-response c-Jun N-terminal kinase (JNK) pathway, which we recently identified to be induced also by the saturated VLCFAs that accumulate in X-ALD patients.^4^ Why IL-15 negatively associates with brain lesion severity (MRI Loes score) in childhood/adolescent CALD patients remains to be investigated, but probably indicates IL-15 as an early marker for the initiation of CALD lesions. Besides IL-15, also IL-12p40 and CCL7 were elevated in yet asymptomatic X-ALD children. Importantly, both IL-12p40 and CCL7 are associated with BBB integrity and concomitant immune cell infiltration.^37^^,^^38^ CCL7, released by astrocytes in traumatic brain injury (TBI) models and a possible marker for early BBB dysfunction,^37^ was the only investigated cytokine upregulated in both children and adult CALD patients in our study. Of note, we previously found CCL7 to be induced in X-ALD macrophages when compared with healthy control cells.^4^ Like IL-15, CCL7 is directly linked to JNK activation, further highlighting the importance of this pathway in the context of X-ALD. Finally, we detected increased blood levels of CXCL8 and CCL11 in childhood CALD. Similar to CCL7, CXCL8 is associated with migration, invasion, and JNK signalling.^39^ CCL11 is released by activated astrocytes in the CNS and several different cell types in the periphery^40^ and high blood levels of CCL11 have recently been associated with neurodegenerative processes.^40^

We acknowledge that our study has potential limitations. Firstly, AUC values were calculated on the same data set that was used for estimating the logistic regression models, thus AUC estimates might be over-optimistic. Secondly, the reference samples from both healthy controls and asymptomatic X-ALD children were derived from people that lacked clinical manifestations of other somatic diseases associated with increased plasma NfL levels such as metabolic disorders.^41^ These subclinical disease conditions can occur with similar prevalence in X-ALD patients. However, our NfL-based CALD risk assessment has not been corrected for such possible comorbidities. Thirdly, despite strong association between plasma and serum NfL measurements and slight but not statistically significant differences in univariable analysis between these two sample types,^5^^,^^24^ the proposed NfL cut-off value for risk prognosis of CALD onset has been determined for and, thus, is only applicable to plasma as sample type. Further studies are needed to verify or adjust the plasma-derived CALD indicative cut-off level also for serum samples. Finally, the development as well as the validation cohort consisted of a limited number of samples, especially from asymptomtic children/adolescents with X-ALD. This relatively low number induces a considerable uncertainty for the estimated prognostic capability (accuracy) of 96% calculated for the validation cohort. Thus, further external validation or recalibration of the CALD-discriminating NfL cut-off level based on plasma NfL measurements in prospective cohorts, preferentially derived from newborn screening approaches, is needed to fully establish its clinical utility.

In conclusion, blood-based biomarker risk prediction integrating plasma NfL measurement is useful to indicate CALD in childhood/adolescent X-ALD patients aged 4–18 years. In future settings, it may offer an improved decision support tool to stratify children or adolescents with X-ALD by their risk for cerebral involvement, thus identifying those patients for MRI analysis who may need timely therapeutic interventions such as HSCT or gene therapy.

## Contributors

IW, JB and PR conceptualised and designed the study. IW, JB, MP, PWS, SF, KGP, MCR, JG, AH, HAY, EM, CC, VC, SFP, PA, AB, ME, FE, TL, AP, CGB, WK and JSK collected data. IW, JB, BZ and SFP reviewed the literature. AG performed the statistical analysis. IW analysed the data and drafted the manuscript. IW, JB, SFP and BZ did the critical revision. IW and JB accessed and verified the underlying data reported in this manuscript. All authors contributed to data interpretation and read and approved the final manuscript.

## Data sharing statement

All data are included in the manuscript or in the Supplementary Materials and are available from the corresponding authors upon request with publication of this manuscript. Source data are provided with this paper.

## Declaration of interests

MP received support from Amicus, Merck, Novartis and Sanofi-Genzyme; BZ received support from ACTRIMS 2022 and 2023 endMS SPRINT; JG received support from Quanterix; HAY was supported by an emerging investigator grant from ALD connect; CGB received grants from the German Research Foundation and the 10.13039/501100010570Ministry for Science and Culture of Lower Saxony; ME received support from Minoryx and is member of the advisory board of Minoryx, Poxel and SwanBio Therapeutics; FE is holding a license for “Intrathecal delivery of nucleic acid sequences encoding ABCD1 for treatment of Adrenomyeloneuropathy” (NO. 29539-021PCT), received consulting fees from SwanBio Therapeutics and UpToDate, is founder of SwanBio Therapeutics, ALD Connect and organizer of trial sites for ASPA, Bluebird Bio Therapeutics, Ionis Pharmaceuticals and Sanofi; AP received consulting fees from Swanbio Therapeutics and Sanofi and is member of the Advisory Board of Bluebird Bio Therapeutics and MedDay Therapeutics. JSK is member of the advisory board for Krabbe Disease of PassageBio. MCR received a grant from Novartis. EM has received funding from the 10.13039/100000002National Institutes of Health (K23NS118044). All remaining authors declare no competing interests.

## References

[1] Turk B.R., Theda C., Fatemi A., Moser A.B. (2020). X-linked adrenoleukodystrophy: pathology, pathophysiology, diagnostic testing, newborn screening and therapies. Int J Dev Neurosci.

[2] Mosser J., Douar A.M., Sarde C.O. (1993). Putative X-linked adrenoleukodystrophy gene shares unexpected homology with ABC transporters. Nature.

[3] Weinhofer I., Zierfuss B., Hametner S. (2018). Impaired plasticity of macrophages in X-linked adrenoleukodystrophy. Brain.

[4] Zierfuss B., Buda A., Villoria-Gonzalez A. (2022). Saturated very long-chain fatty acids regulate macrophage plasticity and invasiveness. J Neuroinflammation.

[5] Weinhofer I., Rommer P., Zierfuss B. (2021). Neurofilament light chain as a potential biomarker for monitoring neurodegeneration in X-linked adrenoleukodystrophy. Nat Commun.

[6] Wilkinson I.A., Hopkins I.J., Pollard A.C. (1987). Can head injury influence the site of demyelination in adrenoleukodystrophy?. Dev Med Child Neurol.

[7] Weinhofer I., Buda A., Kunze M. (2022). Peroxisomal very long-chain fatty acid transport is targeted by herpesviruses and the antiviral host response. Commun Biol.

[8] Berger J., Forss-Petter S., Eichler F.S. (2014). Pathophysiology of X-linked adrenoleukodystrophy. Biochimie.

[9] Gortz A.L., Peferoen L.A.N., Gerritsen W.H., van Noort J.M., Bugiani M., Amor S. (2018). Heat shock protein expression in cerebral X-linked adrenoleukodystrophy reveals astrocyte stress prior to myelin loss. Neuropathol Appl Neurobiol.

[10] Eichler F.S., Ren J.Q., Cossoy M. (2008). Is microglial apoptosis an early pathogenic change in cerebral X-linked adrenoleukodystrophy?. Ann Neurol.

[11] Bergner C.G., van der Meer F., Winkler A. (2019). Microglia damage precedes major myelin breakdown in X-linked adrenoleukodystrophy and metachromatic leukodystrophy. Glia.

[12] Raymond G.V., Aubourg P., Paker A. (2019). Survival and functional outcomes in boys with cerebral adrenoleukodystrophy with and without hematopoietic stem cell transplantation. Biol Blood Marrow Transplant.

[13] Cartier N., Hacein-Bey-Abina S., Bartholomae C.C. (2009). Hematopoietic stem cell gene therapy with a lentiviral vector in X-linked adrenoleukodystrophy. Science.

[14] Eichler F., Duncan C., Musolino P.L. (2017). Hematopoietic stem-cell gene therapy for cerebral adrenoleukodystrophy. N Engl J Med.

[15] Sailor K.A., Agoranos G., Lopez-Manzaneda S. (2022). Hematopoietic stem cell transplantation chemotherapy causes microglia senescence and peripheral macrophage engraftment in the brain. Nat Med.

[16] Miller W.P., Mantovani L.F., Muzic J. (2016). Intensity of MRI gadolinium enhancement in cerebral adrenoleukodystrophy: a biomarker for inflammation and predictor of outcome following transplantation in higher risk patients. AJNR Am J Neuroradiol.

[17] Albersen M., van der Beek S.L., Dijkstra I.M.E. (2022). Sex-specific newborn screening for X-linked adrenoleukodystrophy. J Inherit Metab Dis.

[18] Moser A.B., Seeger E., Raymond G.V. (2022). Newborn screening for X-linked adrenoleukodystrophy: past, present, and future. Int J Neonatal Screen.

[19] Engelen M., van Ballegoij W.J.C., Mallack E.J. (2022). International recommendations for the diagnosis and management of patients with adrenoleukodystrophy: a consensus-based approach. Neurology.

[20] Mallack E.J., Turk B.R., Yan H. (2021). MRI surveillance of boys with X-linked adrenoleukodystrophy identified by newborn screening: meta-analysis and consensus guidelines. J Inherit Metab Dis.

[21] Khalil M., Teunissen C.E., Otto M. (2018). Neurofilaments as biomarkers in neurological disorders. Nat Rev Neurol.

[22] Kalm M., Bostrom M., Sandelius A. (2017). Serum concentrations of the axonal injury marker neurofilament light protein are not influenced by blood-brain barrier permeability. Brain Res.

[23] Loes D.J., Hite S., Moser H. (1994). Adrenoleukodystrophy: a scoring method for brain MR observations. AJNR Am J Neuroradiol.

[24] Piehl F., Kockum I., Khademi M. (2018). Plasma neurofilament light chain levels in patients with MS switching from injectable therapies to fingolimod. Mult Scler.

[25] Efron B., Tibshirani R.J., Efron B., Tibshirani R.J. (1993). An introduction to the bootstrap.

[26] Reinert M.C., Benkert P., Wuerfel J. (2020). Serum neurofilament light chain is a useful biomarker in pediatric multiple sclerosis. Neurol Neuroimmunol Neuroinflamm.

[27] Powers J.M., Liu Y., Moser A.B., Moser H.W. (1992). The inflammatory myelinopathy of adreno-leukodystrophy: cells, effector molecules, and pathogenetic implications. J Neuropathol Exp Neurol.

[28] Gong Y., Laheji F., Berenson A. (2022). Peroxisome metabolism contributes to PIEZO2-mediated mechanical allodynia. Cells.

[29] Honey M.I.J., Jaspers Y.R.J., Engelen M., Kemp S., Huffnagel I.C. (2021). Molecular biomarkers for adrenoleukodystrophy: an unmet need. Cells.

[30] Benkert P., Meier S., Schaedelin S. (2022). Serum neurofilament light chain for individual prognostication of disease activity in people with multiple sclerosis: a retrospective modelling and validation study. Lancet Neurol.

[31] Preische O., Schultz S.A., Apel A. (2019). Serum neurofilament dynamics predicts neurodegeneration and clinical progression in presymptomatic Alzheimer's disease. Nat Med.

[32] Wang H., Davison M.D., Kramer M.L. (2022). Evaluation of neurofilament light chain as a biomarker of neurodegeneration in X-linked childhood cerebral adrenoleukodystrophy. Cells.

[33] van Ballegoij W.J.C., van de Stadt S.I.W., Huffnagel I.C. (2020). Plasma NfL and GFAP as biomarkers of spinal cord degeneration in adrenoleukodystrophy. Ann Clin Transl Neurol.

[34] Papa L., Ladde J.G., O'Brien J.F. (2022). Evaluation of glial and neuronal blood biomarkers compared with clinical decision rules in assessing the need for computed tomography in patients with mild traumatic brain injury. JAMA Netw Open.

[35] Jabri B., Abadie V. (2015). IL-15 functions as a danger signal to regulate tissue-resident T cells and tissue destruction. Nat Rev Immunol.

[36] Saikali P., Antel J.P., Pittet C.L., Newcombe J., Arbour N. (2010). Contribution of astrocyte-derived IL-15 to CD8 T cell effector functions in multiple sclerosis. J Immunol.

[37] Xue J., Zhang Y., Zhang J., Zhu Z., Lv Q., Su J. (2021). Astrocyte-derived CCL7 promotes microglia-mediated inflammation following traumatic brain injury. Int Immunopharmacol.

[38] Mondal S., Roy A., Pahan K. (2009). Functional blocking monoclonal antibodies against IL-12p40 homodimer inhibit adoptive transfer of experimental allergic encephalomyelitis. J Immunol.

[39] Ha H., Debnath B., Neamati N. (2017). Role of the CXCL8-CXCR1/2 axis in cancer and inflammatory diseases. Theranostics.

[40] Huber A.K., Giles D.A., Segal B.M., Irani D.N. (2018). An emerging role for eotaxins in neurodegenerative disease. Clin Immunol.

[41] Manouchehrinia A., Piehl F., Hillert J. (2020). Confounding effect of blood volume and body mass index on blood neurofilament light chain levels. Ann Clin Transl Neurol.

