# Supplementary material for: Biomarker-based risk prediction for the onset of neuroinflammation in X-linked adrenoleukodystrophy
Source: eBioMedicine. 2023 Sep 7;96:104781. doi: 10.1016/j.ebiom.2023.104781 (PMC10497986; doi:10.1016/j.ebiom.2023.104781)
Supplement: Supplementary Information [file mmc1.docx]

**Supplementary information**

**Weinhofer et al., „Biomarker-based risk prediction for the onset of neuroinflammation in X-linked adrenoleukodystrophy“**

Isabelle Weinhofer^1^*, Paulus Rommer^2^, Andreas Gleiss^3^, Markus Ponleitner^2^, Bettina Zierfuss^1,4,^, Petra Waidhofer-Söllner^5^, Stéphane Fourcade^6^, Katharina Grabmeier-Pfistershammer^5^, Marie-Christine Reinert^7^, Jens Göpfert^8^, Anne Heine^8^, Hemmo AF Yska^9^, Carlos Casasnovas^6,10^, Verónica Cantarín^11^, Caroline G. Bergner^12^, Eric Mallack^13^, Sonja Forss-Petter^1^, Patrick Aubourg^14^, Annette Bley^15^, Marc Engelen^9^, Florian Eichler^16^, Troy C. Lund^17^, Aurora Pujol^6^, Wolfgang Köhler^12^, Jörn-Sven Kühl^18^ and Johannes Berger^1^*

^1^ Department of Pathobiology of the Nervous System, Center for Brain Research, Medical University of Vienna, Vienna, Austria

^2^ Department of Neurology, Comprehensive Center for Clinical Neurosciences and Mental Health, Medical University of Vienna, Vienna, Austria

^3^ Institute of Clinical Biometrics, Center for Medical Data Science, Medical University of Vienna, Vienna, Austria

^4^ Department of Neuroscience, Centre de Recherche du CHUM, Université de Montréal, Montréal, Canada

^5^ Division of Immune Receptors and T Cell Activation, Institute of Immunology, Center for Pathophysiology, Infectiology and Immunology, Medical University of Vienna, Austria

^6^ Neurometabolic Diseases Laboratory, Bellvitge Biomedical Research Institute (IDIBELL), Barcelona, Catalonia, Spain and Biomedical Research Networking Center on Rare Diseases (CIBERER), ISCIII, Madrid, Spain

^7^ Division of Pediatric Neurology, Department of Pediatrics and Adolescent Medicine, University Medical Center Göttingen, Göttingen, Germany

^8^Applied Biomarkers and Immunoassays Working Group, NMI Natural and Medical Sciences Institute at the University of Tübingen, Reutlingen, Germany

^9^ Department of Pediatric Neurology, Amsterdam Public Health, Amsterdam University Medical Center, Amsterdam, The Netherlands

^10^Neuromuscular Unit, Neurology Department, Hospital Universitario Bellvitge, Bellvitge Biomedical Research Unit, Barcelona, Spain

^11^Infant Jesus Children´s Hospital and Biomedical Research Networking Center on Rare Diseases (CIBERER), ISCIII, Madrid, Spain

^12^ Department of Neurology, Leukodystrophy Clinic, University of Leipzig Medical Center, Leipzig, Germany

^13^ Leukodystrophy Center, Department of Pediatrics, Division of Child Neurology, Weill Cornell Medical College, NewYork-Presbyterian Hospital, New York, NY, USA

^14^ Kremlin-Bicêtre-Hospital, University Paris-Saclay, Paris, France

^15^ Department of Pediatrics, University Medical Center Hamburg Eppendorf, Hamburg, Germany

^16^ Department of Neurology, Harvard Medical School, Massachusetts General Hospital, Boston, MA, USA

^17^ Pediatric Blood and Marrow Transplant Program, Global Pediatrics, Division of Pediatric Blood and Marrow Transplantation, MCRB, University of Minnesota, Minneapolis, MN, USA

^18^ Department of Pediatric Oncology, Hematology and Hemostaseology, University Hospital Leipzig, Leipzig, Germany

**Table of contents**

*Supplementary Figure S1:* Longitudinal assessment of plasma NfL before and after conversion to CCALD p. 3 and in asymptomatic childhood/adolescent X-ALD patients

*Supplementary Figure S2:* ROC analysis for sensitivity and specificity of plasma NfL and GFAP to p. 4 discriminate CALD onset.

*Supplementary Figure S3:* Longitudinal assessment of plasma GFAP before and after conversion to p. 5 CCALD and in asymptomatic childhood/adolescent X-ALD patients.

*Supplementary Figure S4:* Luminex bead array measurements of various chemokines and VEGF in the p. 6 blood of X-ALD patients and healthy controls of similar age.

*Supplementary Figure S5:* Longitudinal assessment of cytokines/chemokines in plasma samples of p. 7 X-ALD children before and after the onset of CCALD.

*Supplementary Figure S6:* Blood cytokine levels and their relationship to MRI-based lesion severity in p. 8 X-ALD patients and healthy controls of similar age.

*Supplementary Figure S7:* Blood GM-CSF levels in X-ALD patients and healthy controls of similar age. p. 9

*Supplementary Table S1*: Detailed characteristics of CALD patients p. 10

*Supplementary Table S2:* Detailed characteristics of the X-ALD validation cohort. p. 12

*Supplementary Table S3:* ROC analysis of cytokines/chemokines to discriminate CCALD and p. 12

asymptomatic X-ALD patients.

***Supplementary Figure S1:* Longitudinal assessment of plasma NfL before and after conversion to CCALD and in asymptomatic childhood/adolescent X-ALD patients.**


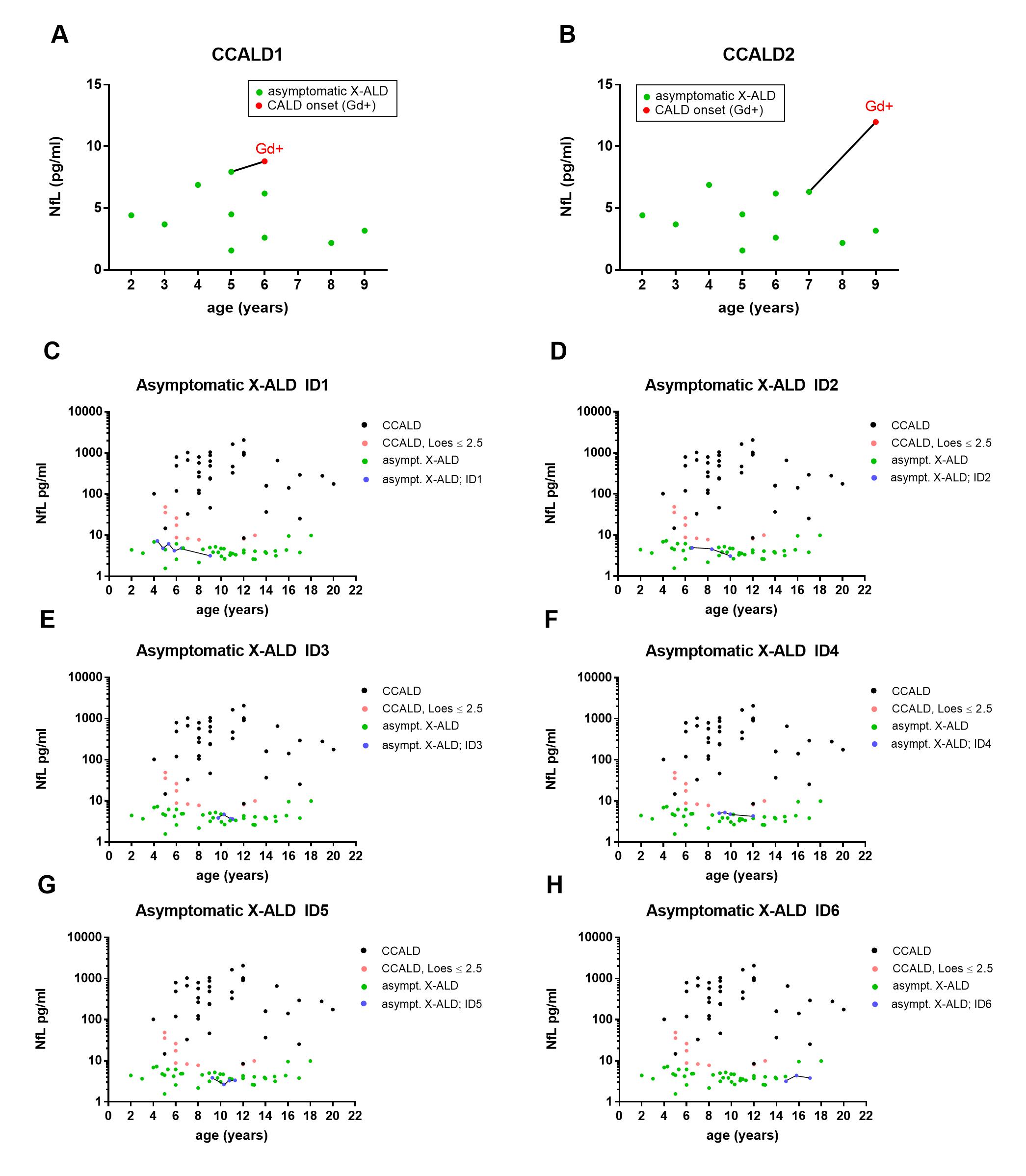


Follow-up of plasma NfL in **(A-B)** two X-ALD patients (CCALD1 and CCALD2) through progression to CALD and **(C-H)** six asymptomatic childhood/adolescent X-ALD patients (Asymptomatic X-ALD ID1 to ID6). Note the logarithmic display in C-H.

***Supplementary Figure S2:* ROC analysis for sensitivity and specificity of plasma NfL and GFAP to discriminate CALD onset.**


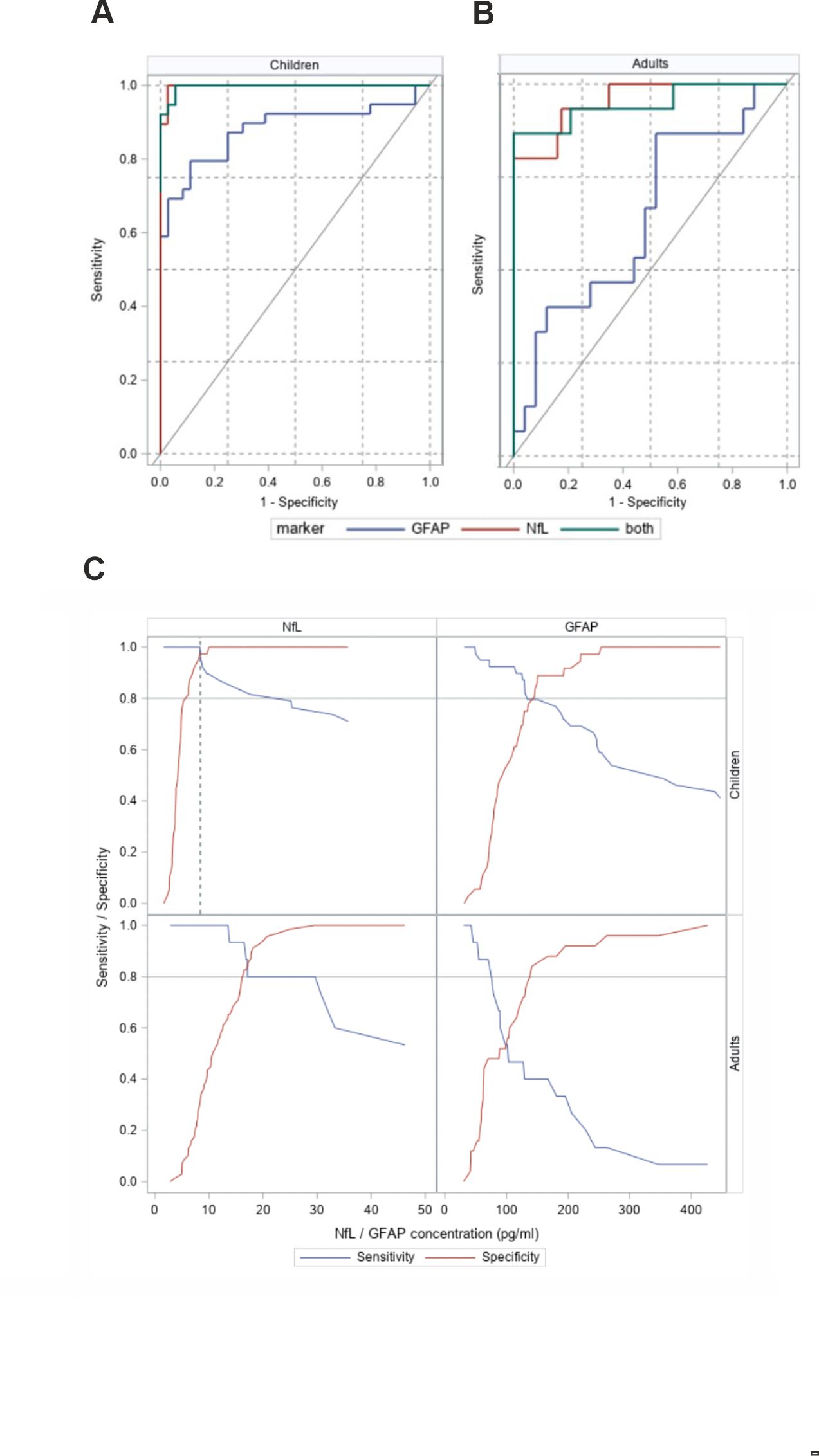


ROC analysis was carried out using plasma samples derived from **(A)** CCALD (NfL, GFAP: *n*=34, total sample number = 36) and asymptomatic X-ALD patients (NfL: *n*=20, total sample number = 40; GFAP: *n*=20, total sample number = 38) and **(B)** ACALD (NfL: *n*=15, total sample number=15; GFAP: *n*=15, total sample number=15) and AMN (NfL: *n*=51, total sample number=69; GFAP: *n*=21 , total sample number=21). NfL, red ROC curve; GFAP, blue ROC curve; NfL and GFAP (model), green ROC curve. **(C)** Sensitivity and specificity for varying cut-off values of NFL and GFAP, respectively, for X-ALD children/adolescents or adults. The vertical dashed line indicates the NfL plasma cut-off value of 8.33 pg/mL yielding an optimal balance between sensitivity and specificity for X-ALD children/adolescents.

***Supplementary Figure S3:* Longitudinal assessment of plasma GFAP before and after conversion to CCALD and in asymptomatic childhood/adolescent X-ALD patients.**


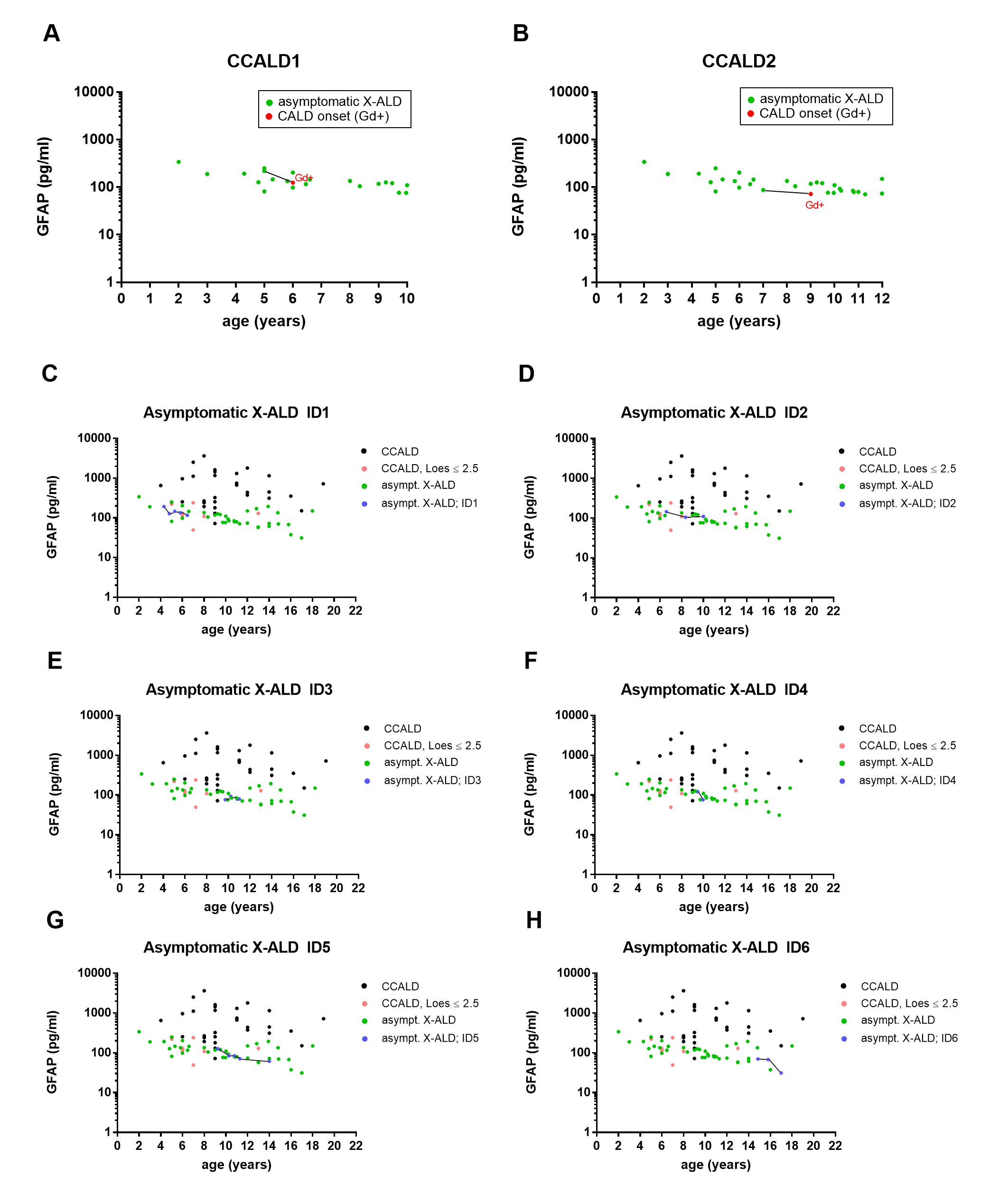


Follow-up of plasma GFAP in **(A-B)** two X-ALD patients (CCALD1 and CCALD2) through progression to CALD and **(C-H)** in six asymptomatic childhood/adolescent X-ALD patients (Asymptomatic X-ALD ID1 to ID6).

***Supplementary Figure S4:* Luminex bead array measurements of various chemokines and VEGF in the blood of X-ALD patients and healthy controls of similar age.**

**
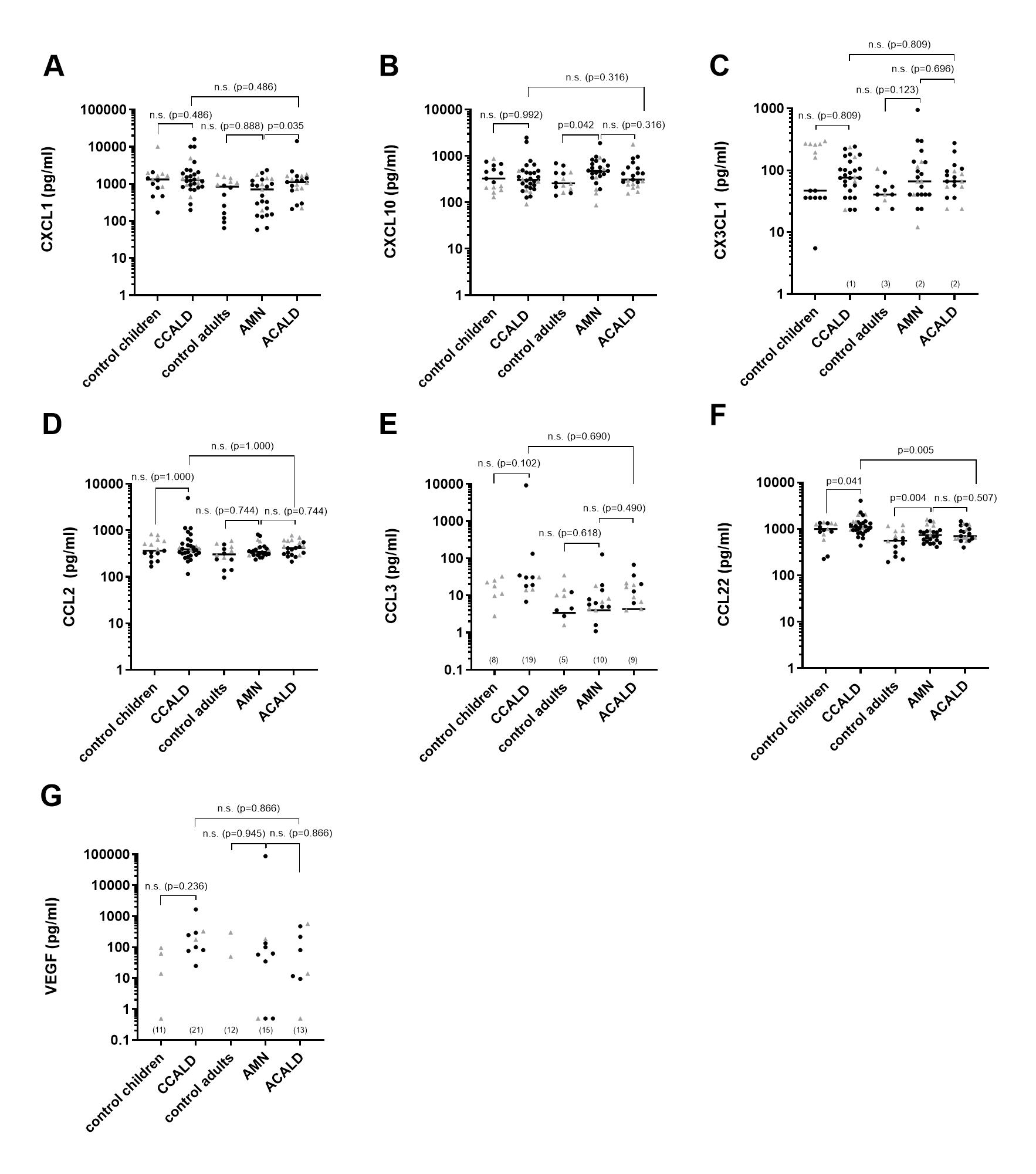
**

The levels of **(A)** CXCL1, **(B)** CXCL10, **(C)** CX3CL1, **(D)** CCL2, **(E)** CCL3, **(F)** CCL22, **(G)** VEGF were determined in plasma and serum samples derived from X-ALD patients (childhood/adolescence CALD [CCALD], *n*=28, median age=9 years, total sample number=31; non-inflammatory AMN, *n*=22, median age=40 years, total sample number=25; adult CALD [ACALD], *n*=19, median age=37 years, total sample number=21) and healthy controls (childhood/adolescent [control children]: *n*=15, median age=11 years, total sample number=15; adult [control adults]: *n*=14, median age=42 years, total sample number=14). Statistical analysis was carried out using a linear mixed model, the median is indicated by a horizontal line. Total sample numbers include samples collected longitudinally from some patients during disease progression. Serum samples are indicated by grey triangles and plasma samples by black circles. The number of data points that were below the detection limit are indicated in brackets above the x-axis.

***Supplementary Figure S5:* Longitudinal assessment of cytokines/chemokines in plasma samples of X-ALD children before and after the onset of CCALD.**


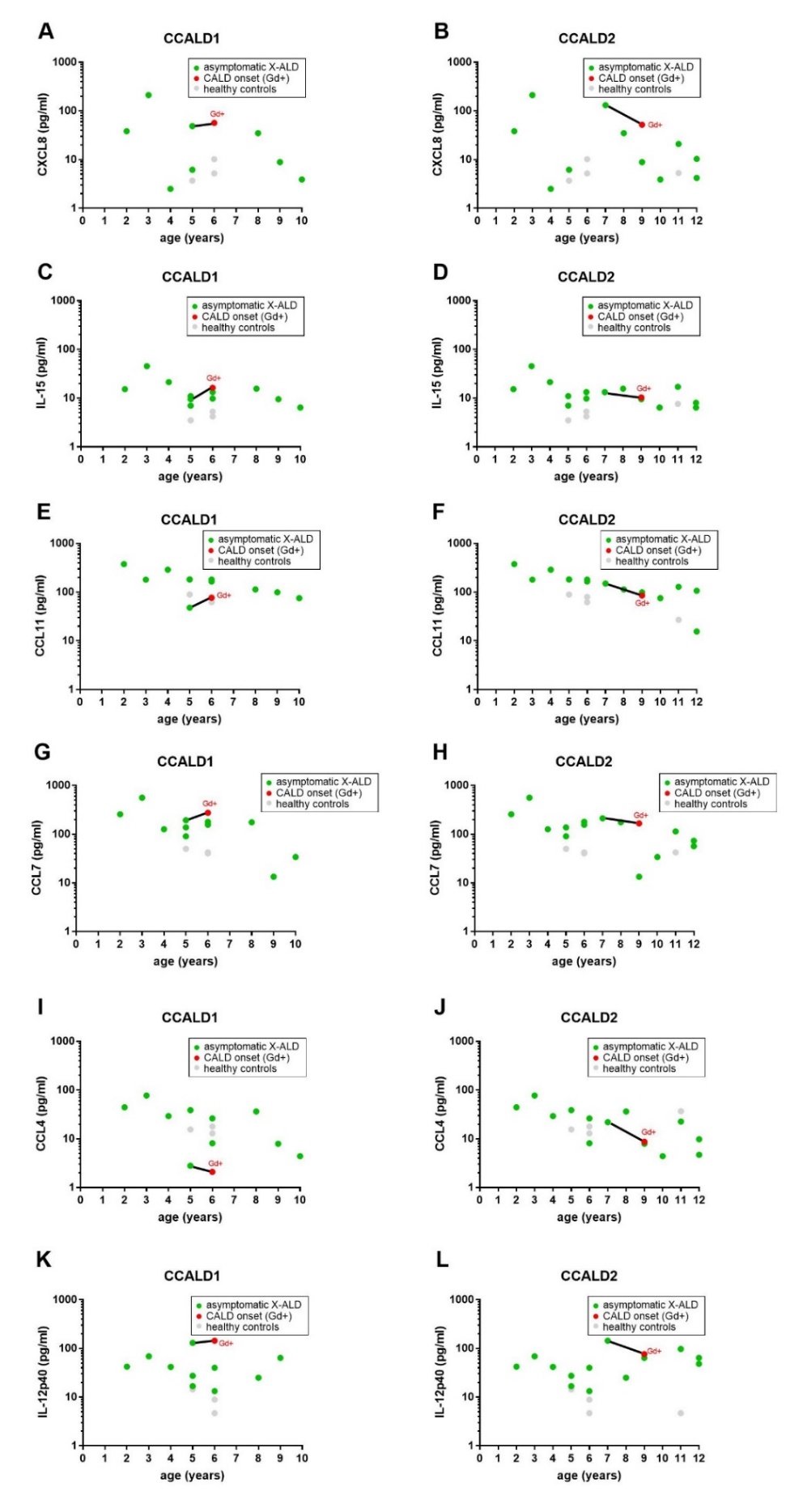


Follow-up of plasma levels of **(A, B)** CXCL8; **(C, D)** IL-15; **(E, F)** CCL11, **(G, H)** CCL7; **(I, J)** CCL4; **(K, L)** IL-12p40 (described in main Fig. 3) in two X-ALD children (CCALD1 and CCALD2) through progression to CALD. The cytokine/chemokine levels in plasma were obtained using the Luminex bead array platform.

***Supplementary Figure S6:* Blood cytokine levels and their relationship to MRI-based lesion severity in X-ALD patients and healthy controls of similar age.**


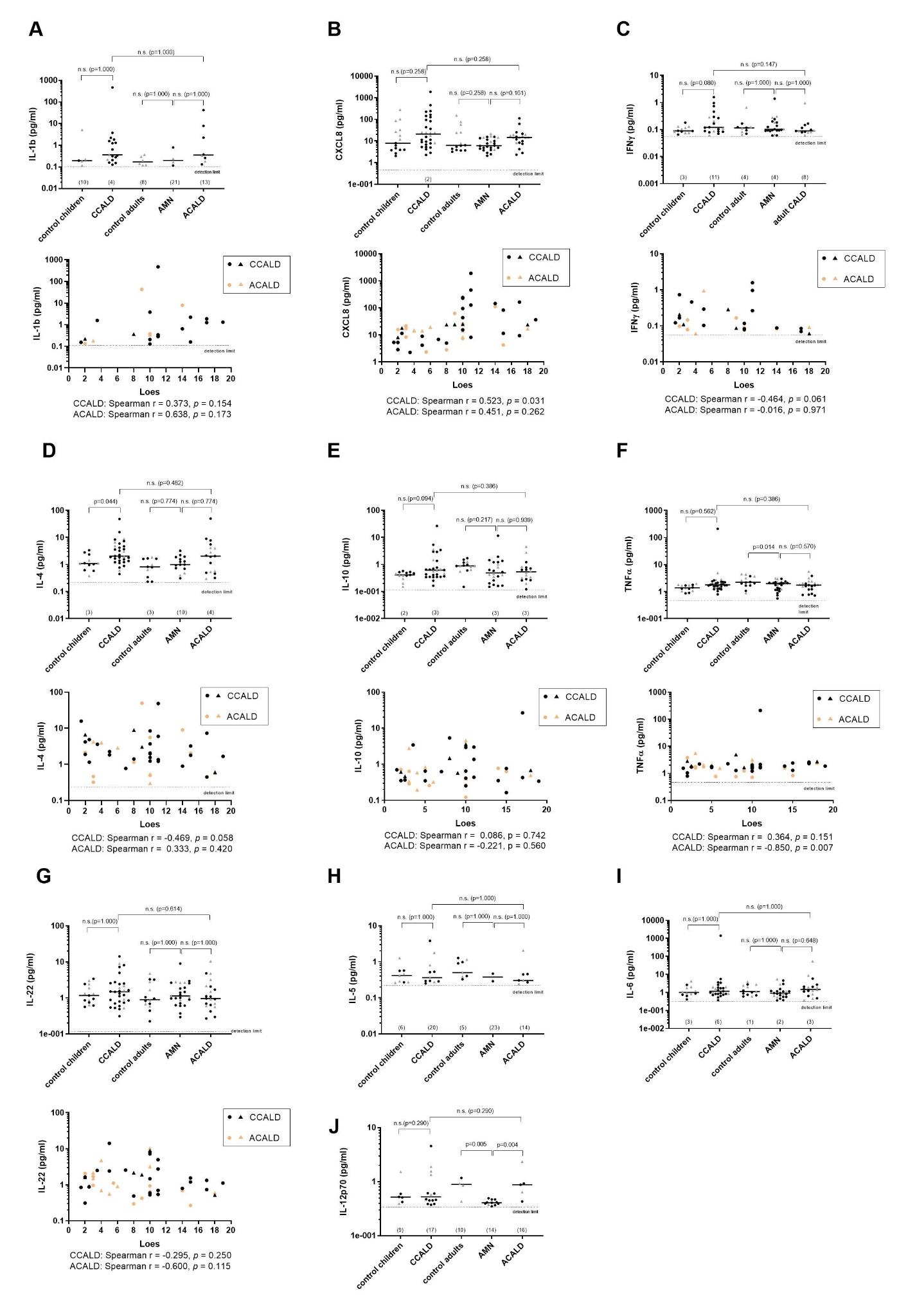


Plasma and serum samples were derived from X-ALD patients (childhood/adolescence CALD [CCALD], *n*=28, median age=9 years, total sample number=31; non-inflammatory AMN, *n*=22, median age=40 years, total sample number=25; adult CALD [ACALD], *n*=19, median age=37 years, total sample number=21), and healthy controls (childhood/adolescent [control children]: *n*=15, median age=11 years, total sample number=15; adult [control adults]: *n*=14, median age=42 years, total sample number=14). Multiplex Simoa was used to determine cytokine levels and association with brain lesion severity by MRI (Loes score) for **(A)** IL-1b, **(B)** CXCL8, **(C)** IFN-γ, **(D)** IL-4, **(E)** IL-10, **(F)** TNFα, **(G)** IL-22, **(H)** IL-5, **(I)** IL-6 and **(J)** IL-12p70. Statistical analysis was carried out using a linear mixed model, the median is indicated by a horizontal line. Total sample numbers include samples collected longitudinally from the same patients during disease progression. Reported Spearman’s *r* have been partialized for the sample type serum or plasma. Serum samples are indicated by grey triangles and plasma samples by black circles. The number of data points that were below the detection limit are indicated in brackets on the x-axis above the respective group.

***Supplementary Figure S7:* Blood GM-CSF levels in X-ALD patients and healthy controls of similar age.**


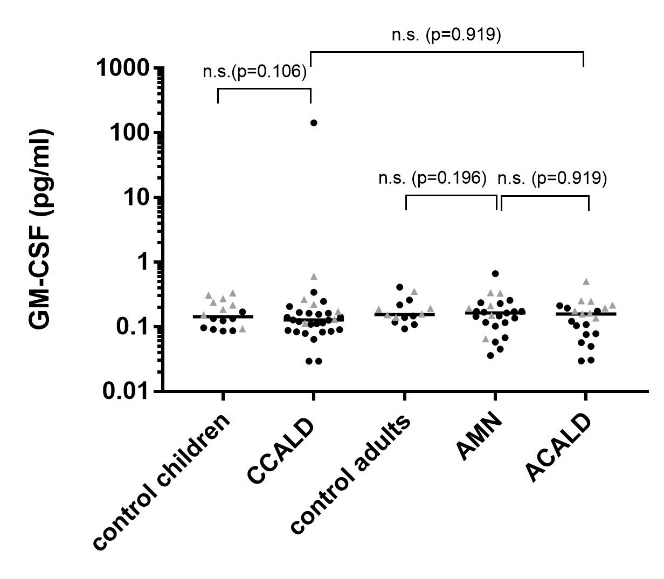


Plasma and serum samples were derived from X-ALD patients (childhood/adolescence CALD [CCALD], *n*=28, median age=9 years, total sample number=31; non-inflammatory AMN, *n*=22, median age=40 years, total sample number=25; adult CALD [ACALD], *n*=19, median age=37 years, total sample number=21) and healthy controls (childhood/adolescent [control children]: *n*=15, median age=11 years, total sample number=15; adult [control adults]: *n*=14, median age=42 years, total sample number=14). The blood levels of GM-CSF were determined by Simoa assay. Statistical analysis was carried out using a linear mixed model, he median is indicated by a horizontal line. Total sample numbers include samples collected longitudinally from the same patients during disease progression. Serum samples are indicated by grey triangles and plasma samples by black circles.

***Supplementary Table S1*:** **Detailed characteristics of CALD patients**

| **ID** | **CALD variant^*^** | **Age (years)**  **at sampling** | **MRI brain lesion severity Loes score^#^** |
| --- | --- | --- | --- |
| 1 | childhood CALD | 6 | 1 |
| 2 | childhood CALD | 9 | N.D. |
| 3 | adolescent CALD | 12 | 11 |
| 4 | adolescent CALD | 11 | 10 |
| 5 | adolescent CALD | 19 | 11 |
| 6 | adolescent CALD | 12 | 11 |
| 7 | adolescent CALD | 16 | 14 |
| 8 | adolescent CALD | 14 | 10 |
| 9 | adolescent CALD | 11 | 10 |
| 10 | childhood CALD | 9 | 8 |
| 11 | childhood CALD | 8 | 18 |
| 12 | childhood CALD | 8 | 2 |
| 13 | childhood CALD | 7 | 2 |
| 14 | childhood CALD | 5 | 2 |
| 15 | childhood CALD | 8  9 | 5  5 |
| 16 | childhood CALD | 6 | 11 |
| 17 | adolescent CALD | 13 | 2.5 |
| 18 | childhood CALD | 8 | 10 |
| 19 | childhood CALD | 8 | 9 |
| 20 | childhood CALD | 6 | 10 |
| 21 | childhood CALD | 9 | 15 |
| 22 | childhood CALD | 8 | 17 |
| 23 | adolescent CALD | 14 | 8 |
| 24 | childhood CALD | 4 | 7 |
| 25 | childhood CALD | 9 | 17 |
| 26 | childhood CALD | 5 | 1.5 |
| 27 | childhood CALD | 5 | 3.5 |
| 28 | childhood CALD | 9 | 19 |
| 29 | adolescent CALD | 11 | 15 |
| 30 | childhood CALD | 7 | N.D. |
| 31 | childhood CALD | 6  6 | 2  2 |
| 32 | adolescent CALD | 14 | 5 |
| 33 | childhood CALD | 9 | 14.5 |
| 34 | adolescent CALD | 12 | 1 |
| 35 | adolescent CALD | 12 | N.D. |
| 36 | childhood CALD | 6 | 3.5 |
| 37 | childhood CALD | 8 | 10 |
| 38 | childhood CALD | 7 | 2.5 |
| 39 | childhood CALD | 7 | 20 |
| 40 | childhood CALD | 9 | 4 |
| 41 | adolescent CALD | 17 | 8 |
| 42 | adult CALD | 47 | 3 |
| 43 | adult CALD | 46 | 3 |
| 44 | adult CALD | 53 | 18 |
| 45 | adult CALD | 43 | 10 |
| 46 | adult CALD | 34 | 3 |
| 47 | adult CALD | 23  25 | 2  3 |
| 48 | adult CALD | 54 | 5 |
| 49 | adult CALD | 51  52 | 4  6 |
| 50 | adult CALD | 31 | 4 |
| 51 | adult CALD | 28 | 10 |
| 52 | adult CALD | 28 | 10 |
| 53 | adult CALD | 48 | 14 |
| 54 | adult CALD | 58 | 15 |
| 55 | adult CALD | 32 | N.D. |
| 56 | adult CALD | 50 | N.D. |
| 57 | adult CALD | 23 | 5.5 |
| 58 | adult CALD | 37 | 9 |
| 59 | adult CALD | 29 | 10 |
| 60 | adult CALD | 27 | 8 |
| 61 | adult CALD | 36 | 10 |
| 62 | adult CALD | 51 | 10.5 |
| 63 | adult CALD | 62 | N.D. |

*Childhood CALD, age onset < 11 years; adolescent CALD, age onset 11 – 20 years; adult CALD, age onset ≥21 years.

^#^Loes score, brain MRI severity scoring system applying a point system ranging from 0 to 34 based on both location and extent of cerebral demyelination as well as atrophy of the brain.

N.D., not determined

***Supplementary Table S2:* Detailed characteristics of the X-ALD validation cohort**.

| **ID** | **Age (years)**  **at sampling** | **NfL**  **(pg/ml)** | **GFAP**  **(pg/ml)** | **Assigned phenotype based on NfL** | **Validated phenotype** | **Loes score^*^** | **Gd-enhancement^#^** |
| --- | --- | --- | --- | --- | --- | --- | --- |
| Val1 | 4.27 | 94.12 | 263.47 | CALD | CALD | 4 | positive |
| Val2 | 4.43 | 638.86 | 857.05 | CALD | CALD | 11.5 | positive |
| Val3 | 4.52 | 3.90 | 84.96 | asymptomatic | asymptomatic | - | - |
| Val4 | 4.59 | 10.33 | 218.52 | CALD | CALD | 1.5 | positive |
| Val5 | 4.75 | 3.52 | 169.73 | asymptomatic | asymptomatic | - | - |
| Val6 | 4.81 | 50.36 | 282.88 | CALD | CALD | 3 | positive |
| Val7 | 5.18 | 74.03 | 585.71 | CALD | CALD | 1 | positive |
| Val8 | 5.34 | 38.09 | 230.20 | CALD | CALD | 3 | positive |
| Val9 | 5.4 | 3.75 | 43.30 | asymptomatic | asymptomatic | - | - |
| Val10 | 5.43 | 17.52 | 97.25 | CALD | CALD | 3 | positive |
| Val11 | 5.74 | 17.99 | 203.68 | CALD | CALD | 1 | positive |
| Val12 | 5.85 | 1037.71 | 4579.57 | CALD | CALD | 12.5 | positive |
| Val13 | 5.9 | 9.42 | 160.86 | CALD | CALD | 1 | negative |
| Val14 | 6.46 | 51.28 | 431.24 | CALD | CALD | 1 | positive |
| Val15 | 6.66 | 39.71 | 33.54 | CALD | CALD | 6 | positive |
| Val16 | 6.91 | 438.65 | 1568.26 | CALD | CALD | 13 | positive |
| Val17 | 6.9 | 4.78 | 280.12 | asymptomatic | asymptomatic | - | - |
| Val18 | 7.95 | 555.33 | 982.81 | CALD | CALD | 11 | positive |
| Val19 | 8.08 | 5.64 | 277.85 | asymptomatic | asymptomatic | - | - |
| Val20 | 8.27 | 10.39 | 129.62 | CALD | CALD | 1 | negative |
| Val21 | 9.21 | 363.41 | 1339.74 | CALD | CALD | 11 | positive |
| Val22 | 9.68 | 14.87 | 51.55 | CALD | CALD | 7 | positive |
| Val23 | 10.63 | 143.83 | 603.65 | CALD | CALD | 20.5 | positive |
| Val24 | 12.21 | 452.05 | 1757.64 | CALD | CALD | 17 | positive |
| Val25 | 12.91 | 3.54 | 24.10 | asymptomatic | CALD | 2 | positive |

* Loes score, brain MRI severity scoring system applying a point system ranging from 0 to 34 based on both location and extent of cerebral demyelination as well as atrophy of the brain.

^#^ Gadolinium (Gd) enhancement on brain MRI indicating alterations of the blood-brain barrier.

***Supplementary Table S3:* ROC analysis of cytokines/chemokines to discriminate CCALD and asymptomatic X-ALD patients.**

| **Cytokine/chemokine** | **AUC (IQR)** |
| --- | --- |
| IL-12p40 | 0.725 (0.562-0.858) |
| CCL4 | 0.688 (0.530-0.830) |
| CCL11 | 0.638 (0.482-0.775) |
| CXCL8 | 0.620 (0.399-0.816) |
| IL-15 | 0.618 (0.461-0.768) |
| CCL7 | 0.333 (0.119-0.688) |
